# Mapping gender role stress scales utilities: a scoping review approach

**DOI:** 10.3389/fpsyg.2024.1436337

**Published:** 2024-09-06

**Authors:** Aitana Aguilera, Laura Villanueva-Moya, Francisca Expósito

**Affiliations:** Mind, Brain and Behavior Research Center (CIMCYC), Department of Social Psychology, University of Granada, Granada, Spain

**Keywords:** gender role stress, scooping review, gender roles, feminine gender role stress, masculine gender role stress

## Abstract

**Introduction:**

Gender role stress emerges as a concept to try to explain the health difficulties presented by men and women due to gender socialization. Thus, gender role stress arises when individuals feel stressed due to their perceived inability to fulfill the demands of their gender role, or when they believe that a particular situation necessitates behavior traditionally attributed to the opposite gender. To evaluate the presence of gender role stress in individuals, two scales were developed: the masculine gender role stress scale and the feminine gender role scale.

**Objective:**

To identify the main thematic areas studied in the behavioral sciences with the feminine gender role stress scale (FGRSS) and the masculine gender role stress scale (MGRSS) as main variables, specifically examining their contributions to the understanding of the attitudes and behaviors of individuals who are affected by gender role stress. We also aimed to analyze the difference, both quantitatively and qualitatively, in terms of scientific literature produced between the scales.

**Method:**

We followed the preferred items for systematic reviews and meta-analyses (PRISMA) checklist. A scoping review of the literature was conducted using systematic techniques, resulting in the inclusion of 87 articles utilizing either of the two scales.

**Results:**

80% (*n* = 72) of the articles employed the MGRSS, while 20% (*n* = 18) utilized the FGRSS. The MGRSS articles were also the most frequently cited in the literature. The FGRSS has been predominantly used to examine the implications for women’s well-being, whereas the MGRSS has primarily been employed to predict disruptive behaviors in men.

**Conclusion:**

This scoping review highlights disparities in the scientific literature concerning the examination of feminine and masculine gender role stress and its consequences for people. Specifically, it points out the limited investigation into feminine gender role stress and its ramifications compared to masculine gender role stress. These findings indicates the lack of a gender perspective even in research intended to study it, and outline the importance of more research with a gender perspective where women are the aim of study.

## 1 Introduction

Gender is interpreted as a social schema through which people learn to behave based on the expectations that society attributes to women and men (Pryzgoda and Chrisler, 2000; Villanueva-Moya and Expósito, 2020). According to the social role theory (Eagly, 1987; Eagly and Wood, 2012), due to physical differences between men and women, there is a social division of tasks by gender: Care behaviors are associated with women (caregivers) and strength behaviors with men (breadwinners). The result of this differentiation is that gender roles assign to women behaviors that foster positive interpersonal relationships, self-silencing, and caring for others; meanwhile, for men, the social expectation consists of being assertive and decision-making behaviors (Brody et al., 2014). These social expectations due to gender socialization have consequences on men and women. Mayor (2015) demonstrated that women suffer disadvantages in health and stress based solely on socio-cognitive explanations. He specifically proved that socialization, particularly through gender roles and gender traits, has been related to the stress process, the experience of stress, and to the health of individuals. Likewise, Mommersteeg et al. (2024) have shown that having a higher score on gender role norms was related to more depressive symptoms, anxiety and psychological stress. Especially in women, depressive and anxious symptoms were significantly higher than in men. On these issues, research in 2020 showed that two thirds of the Spanish population believed that equality between men and women has already been achieved (Statista, 2020). However, recent studies have shown that gender stereotypes are still active in society (Dallimore et al., 2024; Moya and Moya-Garófano 2021). Despite these striking differences, some statistics show that people do not seem to recognize the existing inequality (Statista, 2020), hence the importance of focusing on gender roles and how these norms influence people’s behavior and perceptions.”

From childhood, individuals are socialized to exhibit behaviors and traits aligned with their gender roles, making the repercussions of deviance apparent (Bussey and Bandura, 1992). Therefore, despite the persistent socialization pressures, the expectations associated with gender roles are demanding, causing challenges for both women and men to consistently conform to gender roles (Pleck, 1981, 1995). An example of the implications of these socialization pressures can be found in the emotional expression of individuals. Individuals’ emotional expression is shaped by modeling, expectations, reinforcement, and traditional social norms. Brody (1993) explained that socialization differences might be the most proximate and direct cause of gender differences in emotional expressiveness. These socialization differences are influenced by cultural and historical factors and may be perpetuated through a process wherein each generation identifies with and internalizes the values, behaviors, and parenting styles of previous generations. As children internalize these norms and expectations their emotional expression is shaped based on gender norms (Chaplin, 2015). When they reach adolescence, they already witness significant differences in their emotionality as a result of this socialization (Chaplin and Cole, 2005). These differences are maintained in adulthood, women show a greater emotional expression, particularly of positive emotions and internalize more negative emotions, mainly sadness and anxiety. Adult men, on the other hand, express a greater presence of aggressiveness and anger (Chaplin, 2015).” In this line, the gender role strain paradigm (Pleck, 1981, 1995) asserts that the pressures traditional gender roles place on men and women, and the consequences of failing to conform them, usually cause people strain- and stress-motivating feelings, thoughts, and behaviors that can be harmful to themselves and others. Consequently, it is important to analyze the impact of gender roles on individuals, given that gender role socialization influences cognitive appraisal and coping, thereby creating gender differences in vulnerability to particular stressors (Gillespie and Eisler, 1992), and the consequences that this may have for people’s well-being.

There is empirical evidence both for and against the evolution of gender roles (Kelmendi and Jemini-Gashi, 2022; van Well et al., 2005). However, the issue does not lie in whether behaviors are masculine and/or feminine; it is about how gender roles shape a rigid attitude that constrains and dictates individuals’ behaviors and skills, limiting their behavioral repertoire, which sometimes increases their vulnerability to certain health problems. In response to this issue, the concept of gender role stress emerged, explaining the health problems of men and women based on gender socialization. Gender role stress (Eisler and Blalock, 1991; Eisler and Skidmore, 1987; Gillespie and Eisler, 1992; Harrington et al., 2022) refers to the degree to which women and men experience stress in contexts incongruent with their gender roles—for example, when men fail to possess leadership or women fail to be nurturant, which seems to trigger discomfort in both. Based on this premise, individuals may experience stress when they perceive themselves as unable to adhere to the imperatives of masculine/feminine gender roles (Eisler and Skidmore, 1987; Gillespie and Eisler, 1992). Consequently, the emergence of gender role stress may be due to (a) avoiding behaviors that society expects from one’s own gender or (b) performing behaviors assigned to the opposite gender (Eisler and Skidmore, 1987; Gillespie and Eisler, 1992; Kazmierczak, 2010).

As gender roles consider both male and female genders, the concept of gender role stress also applies to both. First, the concept of masculine gender role stress (MGRS; Eisler and Skidmore, 1987) emerged, which refers to the cognitive appraisal of specific situations as stressful for men, including the individual’s thoughts and behaviors. Men experience stress when they consider themselves unable to cope with masculine role imperatives or when they consider that a situation requires unmasculine or feminine behavior (Eisler and Skidmore, 1987). For example, a man might experience masculine gender role stress when his behaviors are more directed toward caring for others than toward achieving his professional goals, deviating from his role as a provider (example of an item related on the MGRS scale: “Staying home during the day with a sick child”). Shortly after the formalization of this construct, Gillespie and Eisler explored whether there was a cognitive-behavioral pattern similar to that of male gender role stress in women. It was found that certain events affected women more than men. Thus, the concept of feminine gender role stress (FGRS; Gillespie and Eisler, 1992) emerged, which refers to the cognitive appraisal of specific situations as stressful for women, including thoughts and behaviors. As with men, women may experience stress when they feel unable to cope with the imperatives of the feminine role or when they feel that a situation requires unfeminine or masculine behavior. For example, a woman may experience feminine gender role stress when her behaviors are more directed toward the achievement of her professional goals than toward caring for others, deviating from her role as a caregiver (example of an item related on the FGRS scale: “Having someone else raise your children”).

In summary, gender role stress appears differentially in men and women. The onset of gender role stress depends on traditional gender and social norms. When individuals attempt to avoid or remain attached to gender norms in their behavior is apparently the triggering factor for the occurrence of gender role stress. While the process of onset is the same for men and women, the social and gender norms surrounding the onset are completely different between men and women.

Currently, researchers agree that MGRS and FGRS negatively affect people’s well-being, with gender role stress being related to eating disorders (Bekker and Boselie, 2002; Martz et al., 1995), alcohol abuse (Klingemann and Gomez, 2010) or the presence of anxiety and depression (Chhabra et al., 2022; Gillespie and Eisler, 1992). Given the importance of all these findings, it is worthwhile to analyze the empirical evidence on these variables. Yet, deviating from the gender role is not without negative consequences. A clear example is the social discrimination women face when they diverge from expected feminine gender roles, known as *backlash* (Brescoll et al., 2018). This discrimination has significant repercussions on women, such as increased stress, tension, and frustration (Burke, 2014).

Now, focusing on how gender roles are measured, previous literature has utilized various instruments to explore the relationship between masculinity–femininity and well-being, including the Bem Sex Role Inventory (Bem, 1974), the personal attributes questionnaire (Spence et al., 1974), the Male Role Norms Scale (Thompson and Pleck, 1986), and the Traditional Egalitarian Sex Role Scale (Larsen and Long, 1988). These instruments assess masculinity–femininity dimensions as self-ascribed personality characteristics. Although associations between masculinity–femininity and health have been found using these instruments (Arrindell et al., 1993; Fischer, 2007; Jakupcak et al., 2003; Parrott et al., 2011), the results may be limited due to three reasons: (a) These instruments define masculinity–femininity in terms of personality traits, (b) they lack consideration of other components of the masculinity–femininity dimension, such as attitudinal and behavioral components (van Well et al., 2005), and (c) some of them include predominantly positive masculine personality traits, which may hinder the identification of links between instrument outcomes and health or well-being problems.

Regarding these reasons, Eisler and colleagues (Eisler and Blalock, 1991; Eisler and Skidmore, 1987; Gillespie and Eisler, 1992) argued that is not the masculine-feminine behavior or coping strategy that acts as a risk factor for health problems, but rather the rigid or maladaptive gender role-determined attitude that decreases the person’s repertoire of behaviors. On this basis, they introduced the previously mentioned gender role stress concept and developed two instruments to assess the degree to which men and women perceive certain gender role-related situations as stressful. The first one developed was the masculine gender role stress scale (MGRSS; Eisler and Skidmore, 1987), which evaluates gender role stress regarding masculine behaviors. Four years later, the feminine gender role stress scale (FGRSS; Gillespie and Eisler, 1992) was created to evaluate the stress associated with feminine behaviors.

The MGRSS is a 39-item scale that contains specific situations that elicit stress in relation to perceived failure to meet the standards of MGRS, for example, “being outperformed at work by a woman” (Eisler and Skidmore, 1987). The dimensions of this scale include physical inadequacy, emotional inexpressiveness, subordination to women, intellectual inferiority, and performance failure. The scale validation showed that scores (a) differentiated men and women significantly, (b) did not involve sex-related masculinity measures, and (c) were significantly associated with at least two measures of self-related stress, that is, anger and anxiety (Eisler and Skidmore, 1987).

The FGRSS was designed to analyze women’s tendency to experience stress when faced with threats and challenges to feminine gender role commitments, for example, “Having others believe that you are emotionally cold” (Gillespie and Eisler, 1992). The dimensions of this scale are fear of unemotional relationships, fear of physical unattractiveness, fear of victimization, fear of behaving assertively, and fear of not being nurturant. The FGRSS was validated with the following conclusions: (a) The FGRSS dimensions reflect potential stressors that are particularly salient for women as a result of feminine gender role socialization, (b) as in the MGRSS’s case, FGRSS scores were not related to the personal attributes questionnaire’s measure of femininity (Spence et al., 1974), and (c) FGRSS scores were also related to self-reported depression, but it cannot be used as a predictor of depressive symptomatology (Gillespie and Eisler, 1992).

Over the past 3 decades, interest in gender role stress has been slowly growing across the world, along with the use of these instruments. The accumulating range of gender role stress evidence provided us the opportunity to take stock and cover (almost) everything that has happened since the validation of both scales in 1987 and 1992 as objectively as we can (i.e., using systematic principles, transparency, and openness; see the Method section).

In this research, we utilized a principled approach (using preferred reporting items for systematic reviews and meta-analyses [PRISMA]; Tricco et al., 2018) to review empirical articles using either the MGRSS or FGRSS. This enabled us to synthesize the multidisciplinary empirical literature on the applicability and conclusions derived from the use of the MGRSS and FGRSS. To the best of our knowledge, this will be the first scoping review on the concept of gender role stress. Given that the concept has been little studied, this scoping review could lay the groundwork for future research and increase the visibility of the concept itself. The importance of this review lies in its aim to provide future researchers with an overview of the variables related to gender role stress and to identify gaps in the existing literature, particularly concerning the concept of female gender role stress. Thus, the aim of this review is to identify the main areas of study for each measure (MGRSS and FGRSS), specifically examining how they have contributed to the understanding of attitudes and behaviors; as well as the implications of the findings for understanding individuals’ behavior in relation to the presence gender role stress. Similarly, we aim to analyze the scientific community’s interest with gender role stress. We adopt a bottom-up approach to discover emergent themes from the literature rather than forming *a priori* hypotheses, thereby reducing author bias.

## 2 Materials and methods

### 2.1 Protocol and registration

This scoping review’s pre-registration can be found.^1^ We followed the PRISMA checklist (Tricco et al., 2018). A scoping review of the papers was retrieved using the systematic technique.

### 2.2 Data source

Data were procured from direct consultation and access to the following bibliographic databases in the social and health sciences: MEDLINE (via PubMed), Scopus, and Web of Science. The databases were last revised in February 2024, prior to the writing of this paper.

### 2.3 Information processing

The following search equations were deemed appropriate after consulting the APA thesaurus and MESH terms and finding that the term “gender role stress” is not an indexed term in any platform.

Equation for PubMed: (“gender role stress*” AND ((humans[Filter]) AND (alladult[Filter]))) OR (“gender role stress*”[Title/Abstract] AND ((humans[Filter]) AND (alladult[Filter]))) AND ((humans[Filter]) AND (english[Filter] OR spanish[Filter]) AND (alladult[Filter])).

Equation for Scopus: TITLE-ABS-KEY (“GENDER ROLE STRESS*”) AND (LIMIT-TO (DOCTYPE, “ar”)) AND (LIMIT-TO (LANGUAGE, “English”)).

Equation for Web of Science: (TS = (“GENDER ROLE STRESS”)) AND ((DT==(“ARTICLE”) AND LA==(“ENGLISH” OR “SPANISH”) AND LA==(“ENGLISH”)) NOT (SILOID==(“PPRN”))).

The search equations were maintained using these terms, even though they were not indexed in the thesaurus, to avoid the documentary noise that could have emerged if we used APA thesaurus or MESH terms.

### 2.4 Final selection of articles

Articles that met the following criteria were selected for review:

- Inclusion: articles published in peer-reviewed journals that used the FGRSS (Gillespie and Eisler, 1992), MGRSS (Eisler and Skidmore, 1987), or both.
- Exclusion: (a) systematic reviews, (b) validation trials of new scales, (c) validation trials for the mentioned scales for other populations different from the original scale, (d) articles for which the full text could not be found, (e) articles in languages other than English or Spanish.

Additionally, the bibliography of each selected article was reviewed for possible new papers to be included in the review. The authors of this review evaluated the adequacy of the articles selected. To validate the inclusion of articles, the concordance assessment of the selection (kappa index) had to be greater than 0.60 (Wanden-Berghe and Sanz-Valero, 2012).

### 2.5 Data extraction

**Figure 1:**
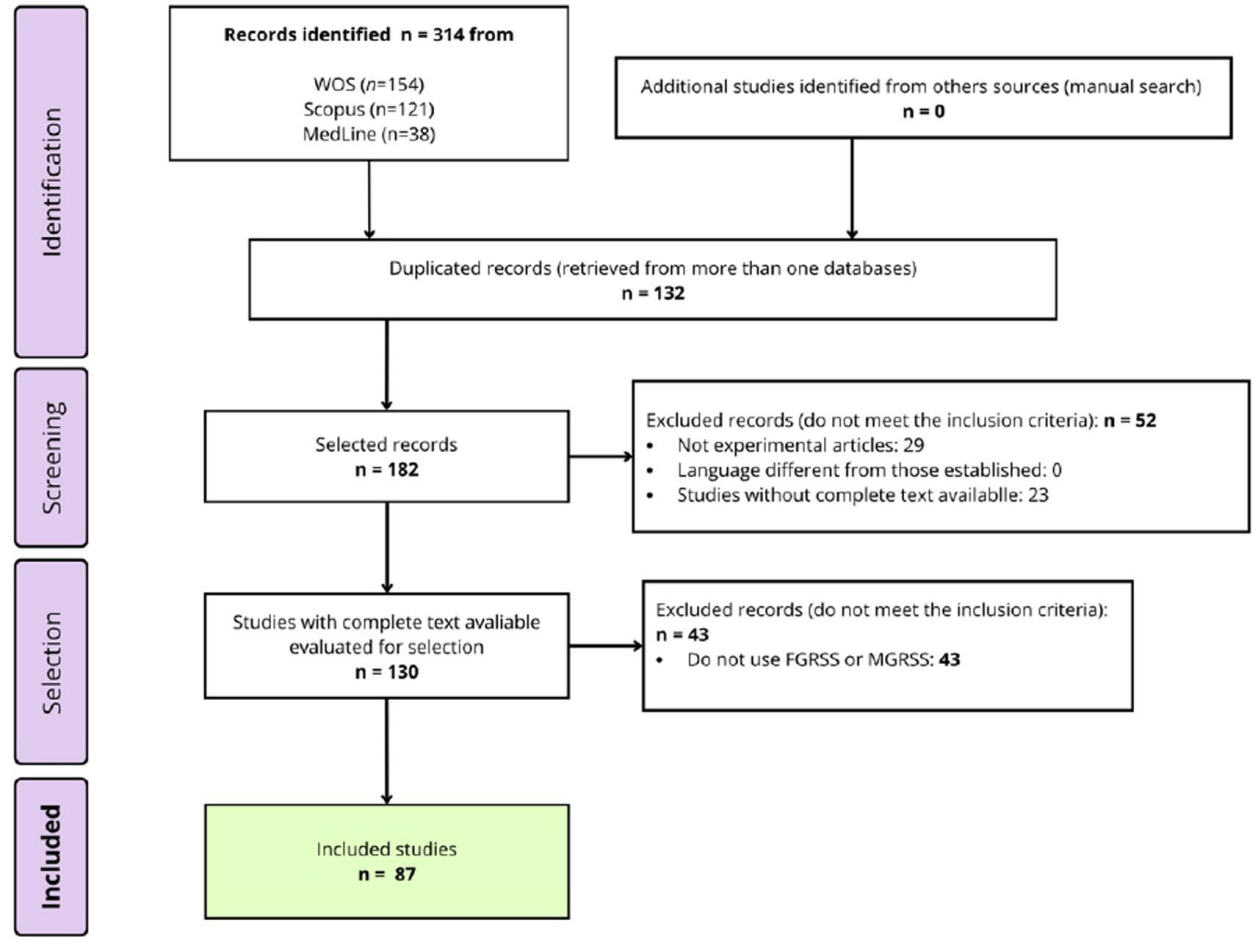
Identification and selection of studies diagram.

Articles were initially categorized based on their use of either the MGRSS or the FGRSS to facilitate comprehension of the results. Subsequently, the multiplatform program ZOTERO, a bibliographic reference manager developed by the Center for History and New Media at George Mason University, was utilized to assign a maximum of 10 tags to each article in both groups. This was done to separate the fields of study addressed with each of the scales. Finally, a critical review was conducted, taking into account the authors, year of publication, abstract of the article, variables studied, scales used, aim of the study, principal results, and authors’ conclusions. For more information about the screening process of the studies selected for the scoping review, see Figure 1. Duplicate records, identified through comparison across multiple databases, were removed using ZOTERO.

## 3 Results

As can be observed in Figure 1, 314 works were retrieved using our search criteria. After screening the 132 duplicate records, 182 final references remained. No additional documents meeting the inclusion criteria were identified through the consultation of bibliographic lists from the selected articles. After applying the inclusion and exclusion criteria, 87 papers were finally selected for the review. For further details, refer to Supplementary Table A1 for FGRSS articles and Supplementary Table A2 for MGRSS articles.^2^

The agreement between the reviewers regarding the appropriateness of the selected studies was calculated using the kappa index, resulting in 98% agreement (*p* < 0.01).

### 3.1 Overall article characteristics

Before starting with the preregistered content analysis of the articles, an exploratory analysis of the scientific literature in the field was carried out. This exploratory analysis was intended to contextualize the level of annual scientific production on gender role stress in the bases used. For this purpose, the raw number of articles found in each database was taken from the search equations (see Figure 1). The data were analyzed with the statistical analysis program R (R Core Team, 2018), specifically the Bibliometrix package (Aria and Cuccurullo, 2017).

**Figure 2:**
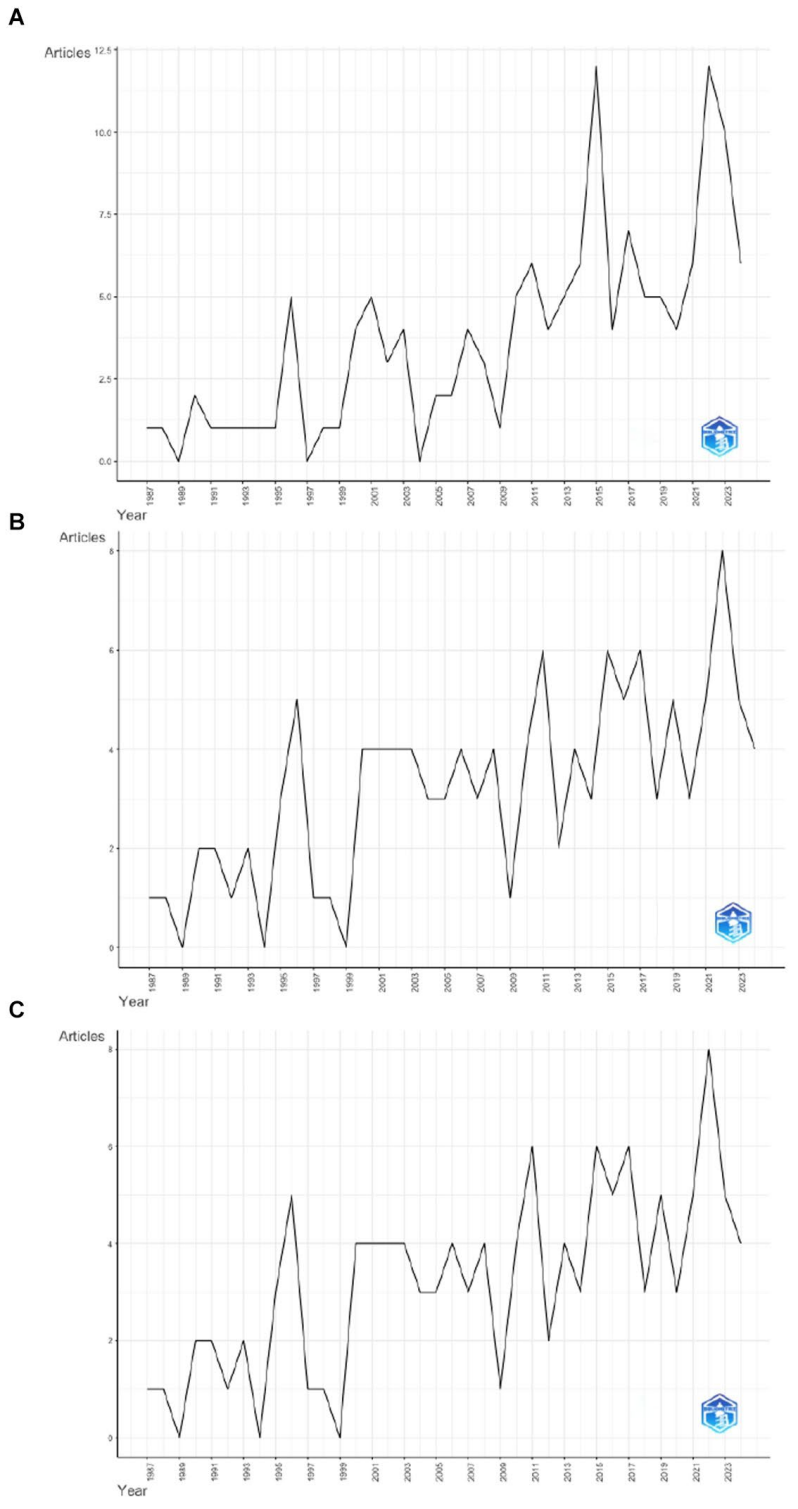
Annual scientific production in different databases concerning FGRS and MGRS. **A** is Web of Science’s line graph, **B** is Scopus’s line graph, and **C** is Medline’s line graph. The horizon axis (years) in the three charts starts in 1987 and ends in 2023. On the vertical axis, the maximum value of articles is 12 for Web of Science **(A)** and eight for both Scopus and MedLine **(B,C)**.

This exploratory review included articles published between 1987 and 2023 on Web of Science, Scopus, and MedLine (see Figure 2).

**Figure 3:**
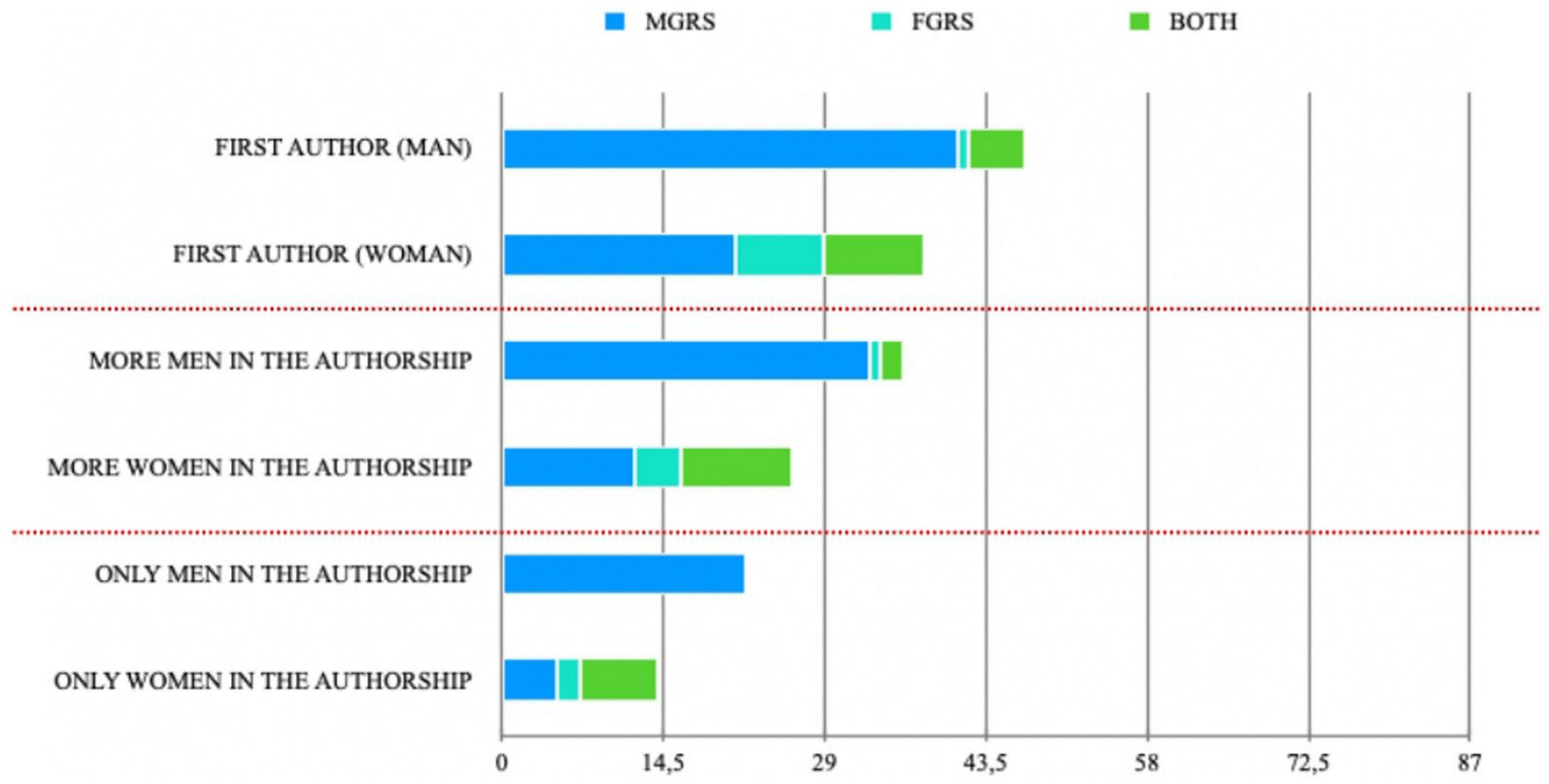
Stocked bar chart about authorship of the papers studied in this paper. On the Y-axis it can be seen the categories studied on this graph separated by dashed red lines, each grouping of two categories should add up to 87. For example, in the case of single authorship by one of the two genres, to the sum of the bars (36), have to add the number of articles that have mixed authorship (47) to reach the total number of articles studied (87). For a clearer understanding of the graph, see Supplementary Table A3.

In the articles included in this review (*n* = 87), in terms of scale usage, 75% (*n* = 65) of the articles used the MGRSS, 10% (*n* = 18) used the FGRSS and 15% (*n* = 13) used both scales. In terms of gender composition, the majority of the articles used male samples (64%), followed by mixed-sample studies (25%), and lastly followed by female-sample use (11%). In this battery of articles, only in the male-sample studies was it specified if participants were heterosexual, veterans, gay/bisexual, substance abusers, or males who batter. Regarding the authorship of the articles included in this study (see Figure 3), it is noteworthy that (a) 54.02% (*n* = 47) of the articles have a male first author, while the remaining 43.68% (*n* = 38) have a female first author. Within these percentages, the distribution of authorship between articles using the MGRSS, FGRSS, or both was more balanced in the female first-authored articles (see Figure 3; Supplementary Table A3). A total of 25 articles had equal numbers of male and female authors. Of the remaining 62 articles, 43.68% (*n* = 36) had more male than female authors. Similar to the previous category, the study of MGRSS, FGRSS, or both was more equitable in articles with a majority of female authors. Finally, about 40% (*n* = 36) of the articles had either male-only (*n* = 23) or female-only (*n* = 14) authorship. Male-only authored articles exclusively studied the MGRSS, while female-only authored articles studied both the MGRSS (*n* = 5), the FGRSS (*n* = 2), and both simultaneously (*n* = 7).

**Figure 4:**
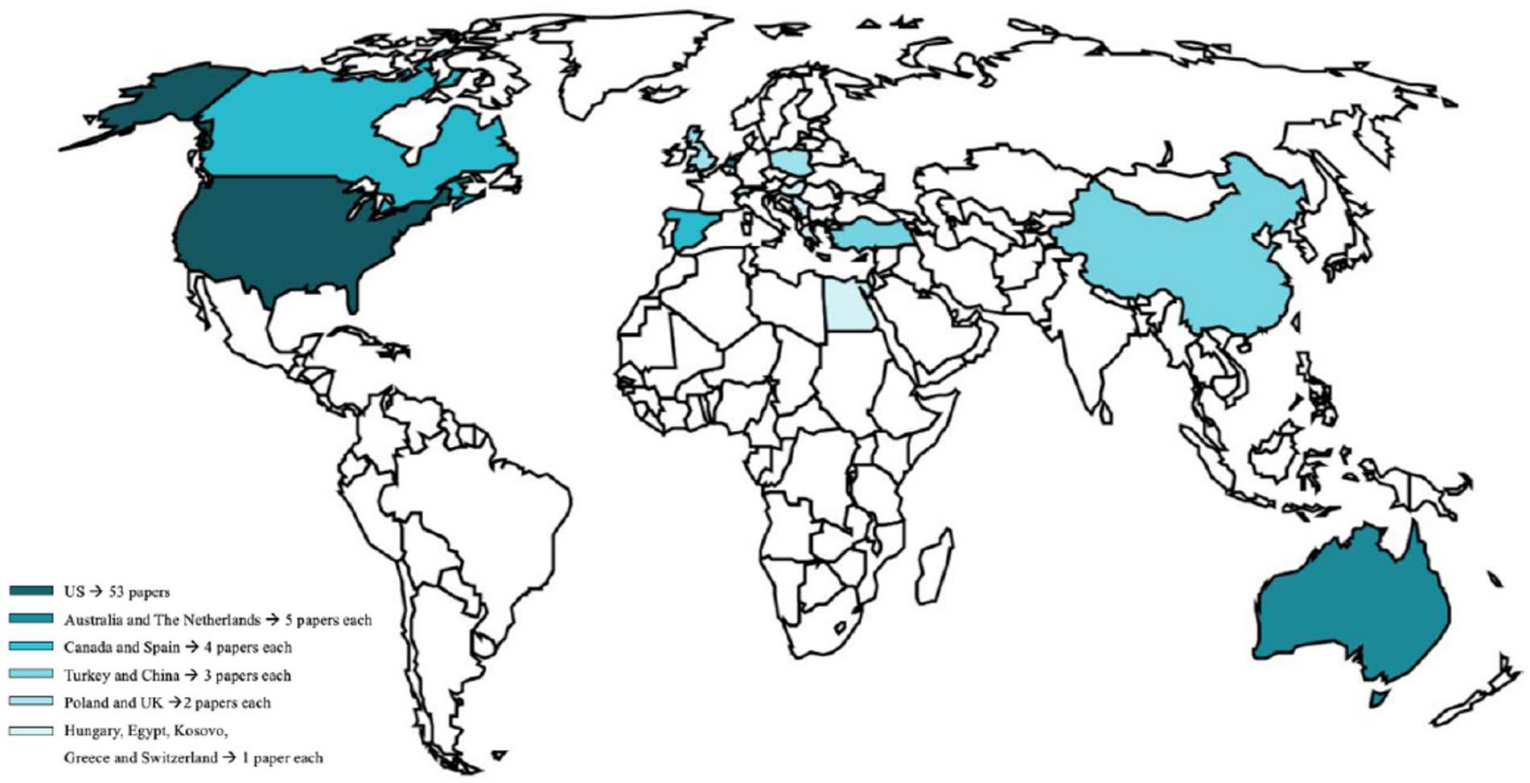
World map infographic of the original country in which studies were conducted. The darker is the country, the higher is the amount of papers published about either FGRS or MGRS.

To conclude the general inspection of the articles included in this review, we analyzed the origin of the articles. As shown in Figure 4, the country with the highest scientific production including the MGRSS and FGRSS scales is the United States (n = 53 papers).

**Figure 5:**
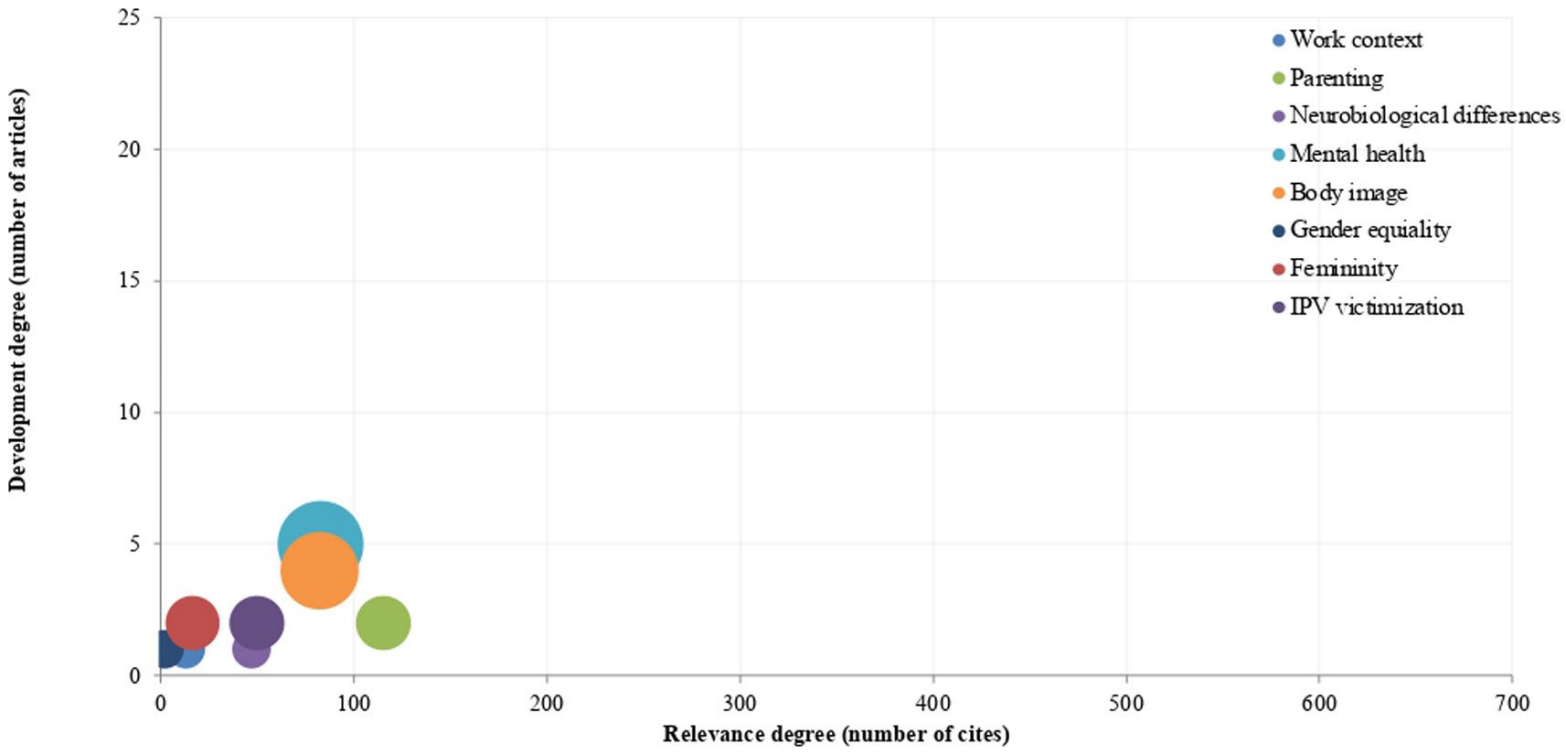
Fields of study on feminine gender role stress. The number of citations has been calculated by averaging the citation counts of each article across the three databases. This average was then combined with the overall averages of articles within the same field to determine the total number of citations.

Focusing on the usage of both scales (FGRSS and MGRSS), the differences between the fields that each scale has been used to study are notable, as well as the percentage of scientific production each scale involves. Particularly, the fields of interest that have involved the usage of the FGRSS are (in order of quantity of scientific productions) mental health (27.8%, *n* = 5), body image (22.2%, *n* = 4), intimate partner violence victimization (11.1%, *n* = 2), femininity (11.1%, *n* = 2), parenting (11.1%, *n* = 2), neurobiological differences (5.6%, *n* = 1), work context (5.6%, *n* = 1), and gender equality (5.6% each, *n* = 1; Figure 5).

**Figure 6:**
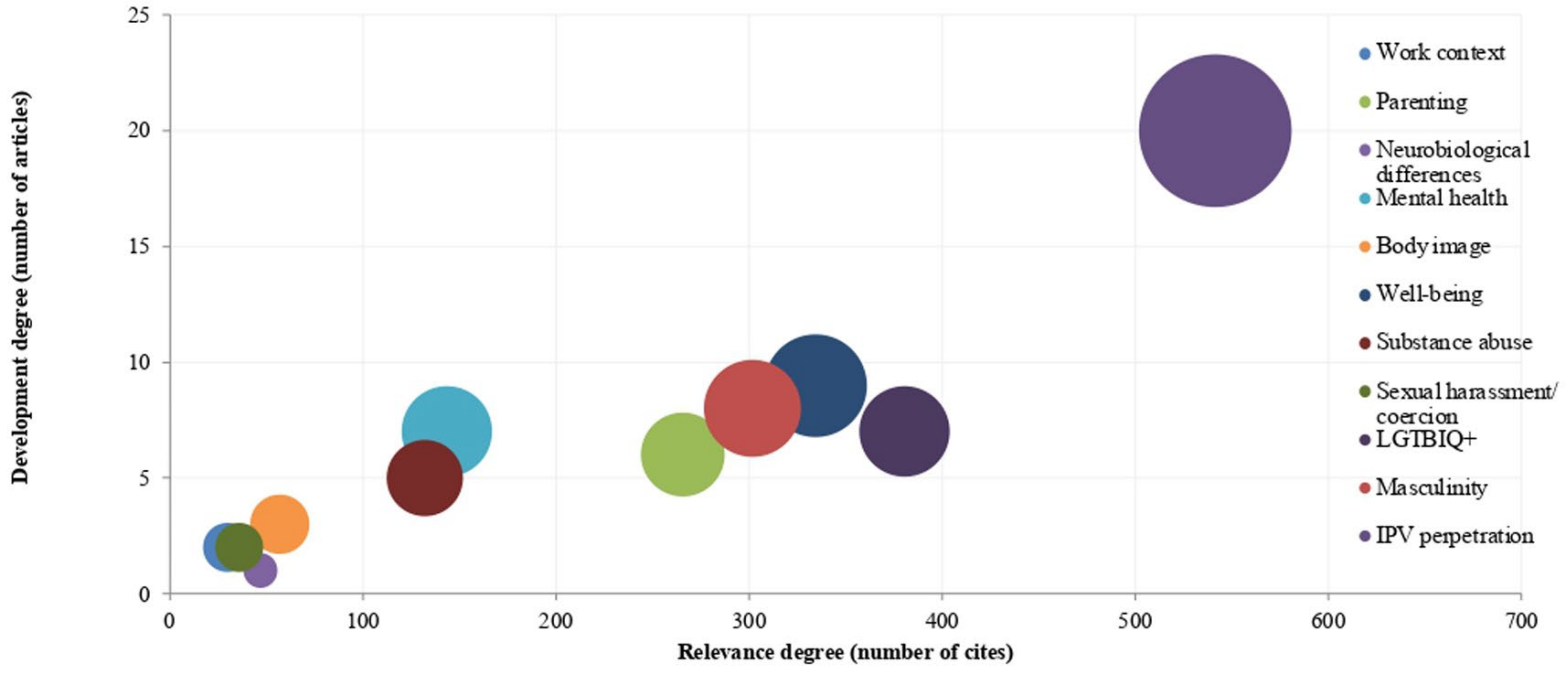
Fields of study on masculine gender role stress. The number of citations has been calculated by averaging the citation counts of each article across the three databases. This average is then combined with the overall averages of articles within the same field to determine the total number of citations.

In the case of the MGRSS, the fields that have been studied are intimate partner violence perpetration (29.9%, *n* = 21), mental health (12.5% each, *n* = 9), well-being (12.5% each, *n* = 9), masculinity (11.1%, *n* = 8), LGBTIQ+ community (9.7%, *n* = 7), parenting (8.3%, *n* = 6), substance abuse (6.9%, *n* = 5), body image (2.8%, *n* = 2), work context (2.8%, *n* = 2), sexual harassment (2.8%, *n* = 2) and neurobiological differences (1.4%, *n* = 1; Figure 6).

Even though the similarity at first sight that can be seen in the percentages, evaluating the numbers of articles behind those percentages is worthwhile. The FGRSS principal field added up to only 12 articles, and the MGRSS principal domains added up to 47 articles (see Figures 3, 4).

Hereupon, the results are structured as follows. The overall domains with gender role stress research appear in separated subsections below (FGRSS and MGRSS) to examine the most remarkable outcomes, the constructs under study, and domain-specific strengths and shortcomings of the research reviewed. Each subsection is concluded by a table containing (a) the main topics of interest that have been used with the tool, (b) the authors who have studied the construct, and (c) the variables related to that tool in the results, summarizing the overall work done in that particular domain. In addition to the tables, each subsection has domain-specific results highlights, limitations, and open theorical questions. Every domain also has a more extended table with the result of each study included (see Supplementary Tables A1, A2).

### 3.2 Feminine gender role stress ( $n_{articles}=18$ )

This domain offers an overview that situates FGRS in the scientific literature from the validation of the FGRSS (Gillespie and Eisler, 1992) thus far. As shown in Table 1, the various domains of interest that have utilized FGRS and the FGRSS include mental health, body image, intimate partner violence victimization, femininity, parenting, neurobiological differences, work context, and gender equality.

**Table 1:** Summary of the variables studied with FGRSS in science.

Topic	Authors	Variables related to FGRS	
Body image	Bekker and Boselie (2002); Kazmierczak and Goodwin (2011); Khattab et al. (2022); Martz et al. (1995).	Pregnancy Masculinity / Androgyny Cultural norms Willing of cosmetic surgery Body image satisfaction	Eating disorders Body dysmorphic disorders Stress (cardiovascular / chronic) Self-esteem Life satisfaction
Femininity / Femininity ideology	Davis et al. (2018); Harrington et al. (2022).	Self-esteem Stress (perceived / racial) Femininity	Strong black women ideology Traditional femininity ideology
Mental health	Berry and Holloway (2022); Efthim et al. (2001); Kargin et al. (2020); Blazek et al. (2013); Kelmendi and Jemini-Gashi (2022); Lopez et al. (2011); Tang and Chung (1997).	Depression Psychological distress Psychological functioning Guilt-proneness Shame-proneness Regret-proneness Borderline personality Histrionic personality	Dependent personality Fear of evaluation Externalization Communal roles Support Empathic concern Gender transformation Women’s sterilization
Work context	Bekker et al. (2001); Birze et al. (2020); Tang and Lau (1996).	Feminine attributes Gender-typed professions	Autonomy Stress
Victimization	Díaz-Aguado et al. (2022); Truman-Schram et al. (2000).	Intimate partner violence Justification of male dominance and violence Traditional female submission	Sexist stereotypes Relationship commitment Risky sexual online behavior Self-esteem
Parenting	Durkin et al. (2001); Morse et al. (2000)	Psychosocial functioning Anxiety Anger	Distress/dysphoria Relationship functioning First-time pregnancy
Neurobiological sex-differences	Lungu et al. (2015).	Brain response Prevalence of depression and anxiety	Hormonal factors Stereotyped gender roles Femininity
Gender equality	Kurtuluş and Bulut (2021).	Fear of negative evaluation Gender equality Egalitarian thinking	

Below, we focus on the main results from each category. See Supplementary Table A1, which provides detailed highlights of results in each domain, separated by authors and organized by year for more information.

#### 3.2.1 Body image

Articles in this domain focus on FGRS vulnerability if body image is perceived as a threat (Martz et al., 1995), eating disorders (Bekker and Boselie, 2002), pregnancy body image (Kazmierczak and Goodwin, 2011), and the relationship between cosmetic surgery and body image (Khattab et al., 2022) in women.

Women who perceive themselves as stressed due to their lack of physical attractiveness (high FGRS) tend to experience more chronic stress (Martz et al., 1995), are more vulnerable to eating disorders (Bekker and Boselie, 2002), are more willing to receive cosmetic surgery, and have lower life satisfaction (Khattab et al., 2022). Furthermore, among pregnant women, body image satisfaction (high FGRS) is negatively related to adhering to cultural norms set for women (Kazmierczak and Goodwin, 2011).

#### 3.2.2 Femininity/femininity ideology

Articles in this domain focus on the relationship between FGRS and African-American women’s identity (Davis et al., 2018) as well as the FGRS, femininity, and self-esteem (Harrington et al., 2022).

Articles involving FGRS and femininity show that, in African-American women, racial stress is more harmful than gender-related stress, especially when women endorse what is known as strong black women ideology versus African-American women who endorse traditional femininity ideology (Davis et al., 2018). Also related to the effects of femininity, Harrington et al. (2022) explained that the negative relation between FGRS and self-esteem has a significant interaction with feelings of femininity.

#### 3.2.3 Mental health

Articles in this domain revolve around psychosexual adjustment (Tang and Chung, 1997), gender-related stress implications in personality and stress (Berry and Holloway 2022; Kargin et al., 2020; Blazek et al., 2013; Kelmendi and Jemini-Gashi, 2022; Lopez et al., 2011), and shame and guilt proneness associated with the FGRSS (Efthim et al., 2001).

The relation between mental health and gender-related stress shows that women have a tendency to experiment higher levels of FGRS when facing a lack of support (Kelmendi and Jemini-Gashi, 2022). The FGRSS also seems to be a predictor of shame and externalization among women (Efthim et al., 2001), and it is negatively associated with self-esteem among women (Kargin et al., 2020).

#### 3.2.4 Work context

This domain focuses on gender-typed professions and how they relate to gender role stress (Tang and Lau, 1996) and stress in the workplace (Bekker et al., 2001; Birze et al., 2020). Compared to men, women feel more FGRS in situations demanding feminine attributes at work. Moreover, in gender-typed professions, people (men and women) experience more gender role stress and burnout (Tang and Lau, 1996). FGRS scores are reduced after attending to stress prevention programs in the workplace (Bekker et al., 2001). FGRS scores in the workplace are positively correlated with emotional labor strategies, indicating that gender role stress is a broader predictor of social distress (Birze et al., 2020).

#### 3.2.5 Intimate partner violence victimization

Articles in this domain focus on the relationship between FGRS and relationship commitment in abusive relationships (Truman-Schram et al., 2000), as well as the consequences for adolescent victims of intimate partner violence concerning self-esteem (Díaz-Aguado et al., 2022).

Articles that explore the relation between FGRS and intimate partner violence victims have shown that there is no FGRSS score difference between women who leave and women who stay in abusive relationships. FGRS is related with relationship commitment; in particular, the “fear of behaving assertively” dimension predicts the likelihood of women reporting what they like about their partner (Truman-Schram et al., 2000). Additionally, adolescent victims of intimate partner violence express less self-esteem and higher FGRS when they do not conform to gender stereotypes (Díaz-Aguado et al., 2022).

#### 3.2.6 Parenting

Articles in this domain revolve around the relationships among FGRS, being a first-time pregnant woman (Morse et al., 2000), and psychosocial functioning of romantic relationships during pregnancy (Durkin et al., 2001).

The relation between first-time pregnancy and gender-related stress shows that women experience more FGRS aligning with a gradual increase in vulnerability from midpregnancy on (Morse et al., 2000). For romantic couples, during pregnancy, mood is strongly related with the levels of gender role stress for both sexes. In women, the variables involved in this psychosocial functioning of the couple are (a) the sense of being cared for by her partner, (b) her satisfaction with the support, and (c) her assessment of the couple’s relationship quality (Durkin et al., 2001).

#### 3.2.7 Neurobiological sex differences

This domain focuses on neurobiological differences between men and women and their possible relation to gender role stress (Lungu et al., 2015). Authors debate whether sex differences in emotion-related connectivity can be fully explained by hormonal factors and by social learning of stereotyped gender roles (as evaluated using the MGRSS and FGRSS).

#### 3.2.8 Gender equality

This domain focuses on gender equality, specifically for women (Kurtuluş and Bulut 2021). Authors concluded that gender equality in women is influenced by mothers’ educational level, FGRS, and fear of negative evaluation. Moreover, they assessed that more egalitarian thinking in women implies and enhances FGRS (Kurtuluş and Bulut 2021).

For an overview of the variables related to FGRS, see Table 1. For a more extended overview of each article’s conclusions, see Supplementary Table A1.

### 3.3 Masculine gender role stress scale ( $n_{articles}=72$ )

This domain offers an overview that situates MGRS in the scientific literature based on the validation of the MGRSS (Eisler and Skidmore, 1987) thus far.

As shown in Table 2, the totality domains of interest that have involved MGRS and the usage of the MGRSS are intimate partner violence perpetration, mental health, well-being, masculinity, LGBTIQ+ community, parenting, substance abuse, body image, work context, sexual harassment, and neurobiological differences.

**Table 2:** Summary of the variables studied with MGRSS in science.

Topic	Authors	Variables related to MGRS	
Body Image	Abbas and Karadavut (2017); Susánszky and Döbrössy (2019); Bekker and Boselie (2002).	Physical appearance Cosmetic surgery Women’s eating disorders	Anxiety Sexual performance Masculinity
Masculinity	Booth et al. (2019); Chadwick and Van Anders (2017); Cohn and Zeichner (2006); Gallagher and Parrott (2011); Gebhard et al. (2019); Jakupcak et al. (2003); Jakupcak (2003); Jakupcak et al. (2005); Leone et al. (2016); Lisco et al. (2015); Reidy et al. (2014); Scaptura and Boyle (2021); Sloan et al. (2015); Smith et al. (2015); Yeung et al. (2015).	Masculinity Emotional control male norm Ambivalence over emotional Expression Expression of anger, hostility and aggression Aggressive / impulsive behavior Fear of emotions	Self-stigma Self-compassion Self-coldness Shame Depressed mood Women’s orgasms function Attraction to guns Psychological well-being
Mental health	Arrindell et al. (1993); Arrindell et al. (2003); Berry and Holloway (2022); Efthim et al. (2001); Harrington et al. (2022); Jakupcak et al. (2006); Kargin et al. (2020); Blazek et al. (2013); Lopez et al. (2011); McCreary et al. (1996); Ragsdale et al. (1996).	Shame proneness Strategy of externalization Guilt proneness Emotional distress Self-esteem Femininity Alexithymia Social support	Fear of emotional states / fears Personality traits Depression Hostility Anxiety Hopelessness Loneliness PTSD symptomatology
Work Context	Bekker et al. (2001); Birze et al. (2020); Tang and Lau (1996); Watkins et al. (1991).	Emotional exhaustion Personal loss Depersonalization Burnout / Stress	Type A behavior Hostility Life dissatisfaction Cardiovascular diseases
IPV and Sexual attitudes	Anderson and Anderson (2008); Chan and Poon (2023); Copenhaver et al. (2000); Díaz-Aguado and Martínez-Arias (2022); Eisler et al. (2000); Franchina et al. (2001); Harrington et al. (2020); Jakupcak et al. (2002); McDermott et al. (2017); McDermott and Lopez (2013); Mellon (2013); Merino et al. (2021); Moore and Stuart (2004); Moore et al. (2008); Zapata-Calvente et al. (2019).	Masculinity Traditional masculine identity Masculine gender role imperatives Antifemininity Aggression toward female intimate partners Aggression against women Hostility toward women. IPV proclivity Aggression Pattern Hostile sexism	Controlling behavior Insecure attachment Relationship power Substance abuse Sexual harassment sexual violence. Self-esteem Anxiety / depression Arousal Gender-relevant situations Bystander decision-making
Parenting	Buist et al. (2003); Casselman and Rosenbaum (2014); Chhabra et al. (2022); DeFranc and Mahalik (2002); Durkin et al. (2001); Morse et al. (2000).	Fatherhood Paternal attachment Paternal depression and anxiety Intergenerational violence Anger Hypermasculine norms	Work–family conflict Marital satisfaction Fears of work disruption / sex performance Affection Social support
Neurobiological differences	Lungu et al. (2015).	Hormonal factors Brain response	Stereotyped gender roles
Well-Being	Arrindell et al. (2003); Arrindell et al. (2013); Cosenzo et al. (2004); Eisler et al. (1988); Eisler and Blalock (1991); Kantar and Yalçın (2023); Lash et al. (2024); Mahalik and Lagan (2001); Morrison (2012); Saurer and Eisler (1990).	Masculinity Gender-relevant instructions Anxious / Hostile personality Self-stigma Self-compassion Seeking psychological help Coping patterns	Phobic / Obsessive-compulsive behavior Social network Commitment Expressiveness Spiritual well-being Health habits Cardiac-related health
LGTBIQ+ community and anti-LGTBIQ+ community	Crawford et al. (2002); Daboin et al. (2015); Parrott (2009); Parrott et al. (2011); Parrott et al. (2008); Vincent et al. (2011); Zamboni and Crawford (2006).	Masculinity Antifemininity norm Anger and aggression toward sexual minorities	Racial discrimination Sexual prejudice Sexual risk-taking
Sexual harassment / coercion	Mellon (2013); Moore et al. (2008).	Subordination to women Women’s gender role violations “Manly” sexual behavior Sexual harassment proclivity	Sexual coercion Psychological aggression Injury to partners Failure (work and sexual domains)
Substance abuse	Gallagher and Parrott (2016); Isenhart (1993); Klingemann and Gomez (2010); Leone et al. (2022); McCreary et al. (1999); McDermott et al. (2010).	Sexual inadequacy Sexual harassment (sober/intoxicated women) Alcohol abuse	Masculinity Job inadequacy Fatherhood PTSD symptom

Here, we focus only on the main results from each category. For more detailed highlights of results in each domain, separated by authors and by year, see Supplementary Table A2.

#### 3.3.1 Body image

Articles in this domain focus on MGRS vulnerability if body image is perceived as a threat to men (Susánszky and Döbrössy 2019), eating disorders in women (Bekker and Boselie, 2002), and cosmetic surgery relation with body image in men (Abbas and Karadavut, 2017).

Women with eating disorders, apart from FGRS, experience MGRS as a relevant source of stress (Bekker and Boselie, 2002). Meanwhile, male populations with higher MGRS are more willing to undergo cosmetic surgery to meet the current idealized male form and have less corporal satisfaction (Abbas and Karadavut, 2017). Furthermore, in overweight/obese men, anxiety related to sexual performance or physical appearance causes a significantly high level of MGRS (Susánszky and Döbrössy 2019).

#### 3.3.2 Masculinity

Articles in this domain focus on masculinity identity (Booth et al., 2019; Chadwick and van Anders 2017; Cohn and Zeichner, 2006; Scaptura and Boyle 2021) and effects of masculinity on attitudes and behavior (Gebhard et al., 2019; Jakupcak et al., 2003, 2005; Yeung et al., 2015).

In masculinity identity articles, the resulting highlights are that (a) high-masculinity identity in men with high MGRS can predict aggressive behavior (Cohn and Zeichner, 2006), (b) women’s orgasms lead men with high MGRS to feel more masculine (Chadwick and van Anders 2017), (c) men with high MGRS are likely to believe that seeking counseling would have a negative impact on their self-worth (Booth et al., 2019), and (d) for men with high MGRS, acceptance threat is positively associated with men’s attraction to men and aggressive reactions to perceived disrespect (Scaptura and Boyle 2021).

Concerning the effects of masculinity on attitudes and behavior articles, the main findings show that (a) high MGRS in men is related with masculine ideology, men’s fear of emotions, anger, depressive mood, shame proneness, external expressions of anger and hostility, and aggression (Jakupcak et al., 2003, 2005), (b) the emotional control masculine norm is associated with poorer psychological well-being, especially when men have high ambivalence over emotional expressions (i.e., high scores on the subscale of emotional inexpressiveness of the MGRSS; Yeung et al., 2015), and (c) there is an association between shame-related threatened masculinity and physical aggression tendencies in men who experience MGRS (Gebhard et al., 2019).

#### 3.3.3 Mental health

Articles in this domain explore psychosocial adjustment and health (Efthim et al., 2001; Jakupcak et al., 2006; Blazek et al., 2013; Lopez et al., 2011; McCreary et al., 1996; Ragsdale et al., 1996) and implications on self-esteem (Harrington et al., 2022; Kargin et al., 2020).

MGRS has negative implications on mental health because it is related to depression (Berry and Holloway 2022), self-reported fears (Arrindell et al., 1993), hostility, and anxiety among men and women (McCreary et al., 1996). It is also a contributor to shame and guilt in men, particularly among those strongly committed to gender schemas (Efthim et al., 2001), but it plays a protective role against emotional dysfunction associated with personal distress (Blazek et al., 2013). In male veterans, MGRS is positively associated with alexithymia (Jakupcak et al., 2006).

It seems that the relation between gender-related stress and self-esteem is not clear. On one side, Kargin et al. (2020) affirmed that gender-related stress in both men and women decreases self-esteem. On the other side, Harrington et al. (2022) affirmed that high MGRS is associated with lower self-esteem in women but not in men.

#### 3.3.4 Work context

Articles in this domain explore the relationship between MGRS and workplace perceptions (Watkins et al., 1991), stress at the workplace (Bekker et al., 2001; Birze et al., 2020), as well as gender-typed professions and how they relate to gender role stress (Tang and Lau, 1996).

MGRS is related to emotional exhaustion and depersonalization for both male and female professionals in gender-typed professions (Tang and Lau, 1996). MGRS scores are related to unfavorable perceptions of the workplace among women but favorable perceptions among men (Watkins et al., 1991). MGRS scores are reduced after attending to stress prevention programs in the workplace (Bekker et al., 2001). MGRS scores in the workplace are positively correlated with emotional labor strategies, indicating that gender role stress is a broader predictor of social distress (Birze et al., 2020).

#### 3.3.5 Intimate partner violence perpetration

Articles in this domain cover the intimate partner violence phenomenon in terms of support by the masculine hegemonic norms (Gallagher and Parrott 2011; Leone et al., 2016; Lisco et al., 2015; McDermott et al., 2017; Reidy et al., 2014; Smith et al., 2015), gender-relevant situations (Eisler et al., 2000; Franchina et al., 2001; Harrington et al., 2022; Jakupcak et al., 2002; Moore and Stuart, 2004), adolescent perpetrations (Díaz-Aguado and Martínez-Arias 2022; Merino et al., 2021), attachment or emotions (Anderson and Anderson, 2008; Copenhaver et al., 2000; Jakupcak, 2003; Mahalik et al., 2005; McDermott and Lopez, 2013; Zapata-Calvente et al., 2019) and even ostracism (Chan and Poon, 2023).

The most consistent finding in this domain is that MGRS is related to intimate partner violence perpetration either as a cause or as a mediator. The main findings surrounding masculine hegemonic norms (antifemininity, status, and toughness) are that (a) their relation with MGRS leads to hostility toward women (Gallagher and Parrott 2011), (b) adherence to the antifemininity norm has been normally related to MGRS due to women’s subordinate positions (Smith et al., 2015), and (c) adherence to toughness and antifemininity norms correlates positively with perpetration of physical aggression toward female intimate partners via MGRS (Lisco et al., 2015). In the sexual domain of intimate partner violence, adherence to toughness and antifemininity norms is associated with confidence in intervening in a sexually aggressive event (Leone et al., 2016). In conclusion, MGRS is related to the likelihood of accepting psychological, physical, and sexual dating violence (McDermott et al., 2017).

In the case of gender-related situations involving women’s behavior, the key findings are that more negative attributions and negative affect can be reported for women’s behavior in gender-relevant situations, especially when men have high MGRS (Eisler et al., 2000). In the case of gender-related situations and men’s behaviors, high MGRS in men is related to more negative attributions to those situations, especially if the situation can be perceived as a female threat. Considering female threats, men with high MGRS endorse verbal aggression toward women (Franchina et al., 2001). In terms of emotion, masculine gender-relevant situations produce more feelings (anger, affect, attributions), especially among men with high MGRS (Moore and Stuart, 2004).

Focusing on specific populations, MGRS predicts dating violence against women and sexual harassment in male adolescents (Díaz-Aguado and Martínez-Arias 2022; Merino et al., 2021). Among substance abusers, men with high MGRS show greater levels of anger and verbally abusive behavior toward their female partners (Copenhaver et al., 2000).

#### 3.3.6 Parenting

Articles in this domain revolve around the relationship between MGRS and romantic couples in which the woman is pregnant for the first time (Morse et al., 2000), psychosocial functioning of couples during pregnancy (Durkin et al., 2001), fatherhood (Buist et al., 2003; Casselman and Rosenbaum, 2014; Chhabra et al., 2022), and parental attachment (DeFranc and Mahalik, 2002).

The relation between first-time pregnancy and gender-related stress shows that a couple’s pregnancy invokes anticipated fears of work disruption and less sexual activity in men. Work- and sex- related fears may mask perceived threats to the male identity in some men (who have higher scores on the MGRSS; Morse et al., 2000). For romantic relationships, during pregnancy, mood is strongly related with the levels of gender role stress in both sexes (Durkin et al., 2001).

Adult males report that their levels of MGRS are related with lesser parental attachment and a greater degree of psychological separation (DeFranc and Mahalik, 2002).

Concerning the relation between MGRS and fatherhood, studies have shown that (a) men high in MGRS have a higher tendency toward postnatal depression and anxiety about the change of their social role (Buist et al., 2003), (b) compared to highly masculine males with rejecting fathers and low self-esteem, some highly masculine males with accepting fathers might experience less MGRS due to their strong self-confidence but are still aggressive because that is part of their belief system regarding how a man should behave (Casselman and Rosenbaum, 2014), and (c) men who strongly adhered to traditional masculine norms may find it difficult to transition to an egalitarian fatherhood and, hence, experience high levels of MGRS (Chhabra et al., 2022).

#### 3.3.7 Neurobiological differences

This domain focuses on neurobiological differences between men and women and their possible relation to gender role stress (Lungu et al., 2015). Authors debate whether sex differences in emotion-related connectivity are fully explain by hormonal factors and by social learning of stereotyped gender roles (as evaluated using the MGRSS and FGRSS).

#### 3.3.8 Well-being

Articles in this domain focus on psychosocial well-being (Eisler et al., 1988; Eisler and Blalock, 1991; Saurer and Eisler, 1990), cardiovascular reaction to stress (Cosenzo et al., 2004; Lash et al., 2024; Morrison, 2012), spiritual well-being (Mahalik and Lagan, 2001), health habits (Arrindell et al., 2003; Arrindell et al., 2013; Sloan et al., 2015), and attitudes toward seeking psychological help (Kantar and Yalçın 2023).

Concerning psychosocial well-being, MGRS predicts negative psychosocial and somatic consequences (Eisler et al., 1988; Saurer and Eisler, 1990). Specifically, Eisler and Blalock (1991) said that excessive commitment to masculine gender norms produces high levels of MGRS, which lead to inflexible and maladaptive coping patterns. When facing these psychosocial drawbacks, MGRS in conjunction with self-stigma and self-compassion were negatively related with seeking psychological help among men (Kantar and Yalçın 2023).

MGRS is related to increased systolic blood pressure (Lash et al., 2024), especially in gender-relevant situations that challenge masculinity (Cosenzo et al., 2004), MGRS is also negatively related to cardiac-related health in PTSD male patients (Morrison, 2012). In terms of general health habits, MGRS is related to poor mental and physical health (Arrindell et al., 2003; Arrindell et al., 2013) and worse health habits (Sloan et al., 2015) in both men and women.

#### 3.3.9 LGTBIQ + community and anti-LGBTIQ + community

Articles in this domain focus on sexual prejudice toward the LGTBIQ+ community (Daboin et al., 2015; Parrott et al., 2008; Parrott et al., 2011; Vincent et al., 2011), aggression toward gay men (Parrott 2009), sexual functioning (Zamboni and Crawford 2006), and self-identification (Crawford et al., 2002).

Men’s self-identification as African-American and gay/bisexual lead to higher levels of self-esteem, HIV prevention self-efficacy, stronger social support networks, greater levels of life satisfaction, and lower levels of MGRS (Crawford et al., 2002). Concerning sexual functioning in this population, racial discrimination, gay bashing, and MGRS may increase the chances that sexual problems develop (Zamboni and Crawford 2006).

Regarding aggression toward gay men, adherence to the antifemininity norm elicits anger and aggression toward gay men among men high in MGRS (Parrott 2009).

Regarding sexual prejudice toward the LGTBIQ+ community, studies present in this review conclude that (a) male gender role norms, particularly the antifemininity norm, are strongly associated with anger in response to male and female sexual minorities. Additionally, sexual prejudice and MGRS are important mediators of these associations (Parrott et al., 2008; Parrott et al., 2011), (b) men high in MGRS respond aggressively to masculinity threats, regardless of whether the threat originates from women or gay men (Vincent et al., 2011), and (c) acceptance of the traditional male role norms of status and toughness, MGRS, religious fundamentalism, and sexual prejudice against lesbians and gay men are higher among Black heterosexual men (vs. White heterosexual men; Daboin et al., 2015).

#### 3.3.10 Sexual harassment/coercion

Articles in this domain explore the relationships among MGRS, sexual harassment (Moore et al., 2008), and coercion (Mellon, 2013).

High levels of MGRS in men are related to (a) failure to perform associated with psychological aggression, (b) appearing physically fit and not appearing feminine associated with sexual coercion, and (c) intellectual inferiority associated with injury to partners (Moore et al., 2008).

Mellon (2013) concluded that self-reported harassment proclivity scores were also positively correlated with MGRS scores that were not thematically related (or were, at most, weakly related) to women’s gender role violations.

#### 3.3.11 Substance abuse

Articles in this domain explore the relationships among MGRS, alcohol abuse (Gallagher and Parrott 2016; Isenhart, 1993; Klingemann and Gomez 2010; Leone et al., 2022; McCreary et al., 1999), and PTSD substance abuse treatment (McDermott et al., 2010).

Concerning the relationship between alcohol abuse and MGRS, literature has concluded that (a) in a sample of male inpatient alcohol abusers, higher MGRS scores were related to higher scores on alcohol abuse (Isenhart, 1993), (b) MGRS is a risk factor for alcohol-related problems among men (McCreary et al., 1999), (c) patients (men in inpatient alcohol programs) who acknowledge men-specific treatment needs suffer significantly more from MGRS and problems with sexuality and fatherhood than patients who are not aware of masculinity issues, (4) men higher in MGRS who are intoxicated (alcohol) are more likely than sober men to select an sexually explicit film in which a woman is intoxicated and less likely than sober men to select a sexually explicit film in which a woman is sober (Leone et al., 2022), and (5) men lower, but not higher, in MGRS receiving an intervention manipulation, relative to control, enacted significantly less alcohol-related physical aggression toward women (Gallagher and Parrott 2016).

McDermott et al. (2010) studied the relationship between MGRS and posttraumatic stress disorder symptom severity among male crack/cocaine-dependent patients in residential substance abuse treatment. In their study, MGRS was found to be significantly associated with posttraumatic stress disorder symptom severity when controlling for other variables previously found to be associated with posttraumatic stress disorder.

For an overview of the variables related to MGRS, see Table 2. For a more extended overview of each article’s conclusions, see Supplementary Table A2.

## 4 Discussion

The aim of this study was to identify the main fields of study for the MGRSS and the FGRSS, specifically examining their contributions to understanding attitudes and behaviors in these fields, and to analyze the scientific community’s interest with gender role stress. The first finding of this review suggests that, compared to other fields of social psychology, gender role stress has been relatively understudied. Since the formalization of masculine and feminine gender role stress and the development of their respective scales, scientific production in this context appears to have been growing slowly (see Figure 2). This progressive but slow growth could be interpreted from different perspectives. A positive interpretation is that this growth reflects a gradual and steady increase in the scientific community’s interest in gender role stress as a concept because it provides answers to pertinent questions posed by researchers over the years. Another positive interpretation could be that the heightened interest in researching gender roles, which have long guided behavior and remained largely unnoticed by the scientific community, signifies a shift in focus toward issues of gender socialization. Conversely, a negative interpretation of this growth could be that it reflects intermittent interest in the construct, marked by fluctuating levels of attention. This could be due to social changes, the emergence of new and more contemporary study concepts, or the aging of the scales, which might have become outdated for assessing today’s society.

Before discussing the results regarding the content of the articles included, it is relevant to reflect on the numbers obtained in this research. First, it is striking to note the significant difference in terms of scientific publications in this area between the United States (*n* = 53) and the rest of the scientific community (*n* = 34; Figure 3). This suggests that US authors have paid the most attention to this construct, as is the case in most of the research.

Within the 87 articles reviewed in this research, a total of 65 articles focused on MGRS and used the MGRSS, whereas only 22 articles addressed FGRS and the study of both concepts together. This discrepancy has implications for the conclusions that can be drawn from the studies, as discussed below. The analysis of works using the FGRSS led to several conclusions. Firstly, feminine gender role stress appears to be a precursor to low self-esteem in women across various situations, such as intimate partner violence victimization, low body satisfaction, and feelings of femininity (Díaz-Aguado et al., 2022; Harrington et al., 2022; Kargin et al., 2020). Secondly, the tendency to experience high FGRS in women seems to be influenced by factors such as maternal educational level and femininity (Kurtuluş and Bulut 2021).

Meanwhile, analyzing the works based on MGRS revealed that (a) the MGRSS has been more widely used than the FGRSS, (b) it has been used to generate conclusions about both men and women, and (c) it has been involved in a wider range of studies across scientific literature in general. Specifically, outcomes related to MGRSS concluded that, in general, (a) MGRS acts as a predictor of aggressive behavior in men in the intimate partner violence context, LGTBQ+ community aggressions, and masculinity threat-related context (Cohn and Zeichner, 2006; Parrott 2009; Scaptura and Boyle 2021; Vincent et al., 2011) and (b) MGRS in men is related to masculine ideology, men’s fear of emotions, anger, depressive mood, shame proneness, external expressions of anger and hostility, and aggression (Jakupcak et al., 2003, 2005). Additionally, (c) MGRS implications for mental health are related to depression, hostility, and anxiety among men and women (McCreary et al., 1996), (d) MGRS can be either a cause or a mediator of intimate partner violence perpetration (Gallagher and Parrott 2011; Leone et al., 2016; Lisco et al., 2015; McDermott et al., 2017; Smith et al., 2015), and (e) MGRS is a risk factor for alcohol-related problems among men (McCreary et al., 1999). As women authors, when we approached this review, we started with the idea that there was a gender bias in the research and in how the study of gender role stress was being approached differently for men and women. Regarding the content of the articles, we can draw the general conclusion that, for men, this construct has primarily been used to explain socially undesirable behavior, serving an exculpatory or justifying function for gender role stereotypes (e.g., predicting or justifying violent behavior) or assessing its influence on well-being. In contrast, for women, the findings have mainly focused on the consequences for their well-being or on justifying behaviors that are harmful to themselves, giving gender role stress an explanatory or confirmatory function (e.g., explaining that victimization in intimate partner relationships is due to their gender role stress). This discrepancy may have led to a gap in the use of this construct to explain a broader range of female behaviors, which, as authors, we believe also contributes to maintaining the differences between men and women and the status quo in today’s society.

Considering these results from a general perspective, 80% of the works examined primarily utilized the masculine construct and scale, demonstrating a broader and more extensive exploration across thematic areas compared to the feminine counterpart. This significant difference in the amount of research conducted on one type of gender stress compared to another also influences the number of conclusions that can be drawn from the studies analyzed. Because FGRS has been less studied, the results of the studies tend to be more general in nature (Martz et al., 1995) or shared with research on MGRS (Bekker and Boselie, 2002; Efthim et al., 2001; Kargin et al., 2020). In contrast, MGRS has received more specific attention in areas such as intimate partner violence (Eisler et al., 2000; Moore and Stuart, 2004; Reidy et al., 2014), parenting (Chhabra et al., 2022), and masculinity (Booth et al., 2019). To conclude the detailed reflection on the results found, it is important to highlight the differences in the areas studied. The MGRS encompasses more areas of interest than the FGRS. Specifically, certain topics have not been studied in relation to FGRS: substance abuse, the implications of gender role stress for physical health, the effect of gender role stress on members of the LGBTIQ+ community, and the relationship and/or implications of gender role stress in situations of sexual harassment. When examining these differences at a more specific level within each area, there remain significant disparities. For instance, while MGRS is addressed in 16 papers on intimate partner violence perpetration, its female counterpart is only explored in two papers on intimate partner violence victimization. When examining the content of these articles, it becomes evident that even within the same subject matter (intimate partner violence), the approaches to studying male and female contexts are markedly distinct. Research on male populations posits hypotheses that rationalize male dominant behaviors; meanwhile, studies focusing on female populations tend to propose hypotheses that justify subordination and victimization behaviors.

This review provides clear evidence of the normalization of gender inequality, including in research in which women occupy an inferior position compared to men. Women have been disadvantaged for years, and the consequences of gender socialization for them have gone unnoticed or poorly studied.

However, this gender inequality seems to be not only found in the studies included in this review. Based on the analysis of the authorship of the articles included in this research (see Supplementary Table A3), several issues can be observed: (a) In general terms, when the authors are mostly men, the main topic of interest appears to be MGRS. (b) Conversely, when the majority of the authors are women, the interest is much more dispersed among studying MGRS, FGRS, or both. (c) When the articles are authored solely by men, we did not find any articles addressing FGRS or both MGRS and FGRS. (d) However, when the authorship is composed solely of women, the articles are spread across MGRS, FGRS, and both. Three main conclusions can be drawn from this analysis of authorship. Firstly, it can be concluded that, in general, there is more interest or attention given to the male construct, despite the fact that gender inequality—partly caused by gender roles and associated stress—has greater and more negative consequences for the female population. Secondly, these data may reflect the current proportion of male and female researchers. Finally, these data indicate the level of interest in women’s issues within today’s society and the scientific community in particular. These findings should prompt the research community to reflect on our interests moving forward, particularly regarding gender role stress and, more broadly, in pursuing more egalitarian research for the female population.

Based on the findings of this scoping review and considering the ongoing evolution of gender roles in contemporary society, it would be necessary to update both the scales and definitions of male and female constructs of gender role stress to generate definitive conclusions about their societal impact. This, together with an equal study of both constructs in both female and male populations by the scientific community.

## 5 Limitations

There are a few limitations worth noting in this research. The main limitation has to do with the generation of the database search equations. “Gender role stress” is not included in the MESH terms or the APA thesaurus, which led the authors to generate search equations with a term not indexed in the thesaurus, with the consequent documentary noise or documentary silence that this entailed in the searches of each database studied.

## 6 Conclusion

This scoping review shows the gap in the scientific literature related to the study of male and female gender role stress and its consequences in the population since the formalization of both constructs in 1987 and 1992, respectively. Specifically, it points out the lack of research on FGRS and its consequences for the female population compared to MGRS. For example, some studies on MGRS use the scale with women to corroborate hypotheses, a practice we have not seen in the opposite direction. Therefore, this review merely reflects that gender inequality is not only present in today’s society but also in the scientific literature.

Studies on gender role stress in women have focused more on highlighting aspects that denote the weakness of women as a social group (mental instability, preoccupation with their image, internalizing behaviors, etc.) while studies on male gender role stress have focused more on using this construct as a mechanism that explains and/or moderates socially negative behaviors in men (mental weakness, lack of coping, weakness, sexual orientation, violent behaviors, aggressiveness, etc.).

The unequal number of studies on men and women, as well as the emphasis on the differentiating role of the construct to highlight consequences for men and women, indicates the lack of a gender perspective even in research intended to study it. These findings outline the importance of more research with a gender perspective where women are the aim of study.

## Data Availability

The data presented in the study are deposited in the open science framework repository, available at https://osf.io/ve5t4/?view_only=95b5ba5fe651429299132015a90e7c45. Further queries should be directed to the corresponding author.

## Footnotes

^1^https://osf.io/ve5t4/?view_only=95b5ba5fe651429299132015a90e7c45

^2^In those tables, the summary of articles is not equal to the total number of articles studied in this work. This discrepancy arises because certain articles covered both MGRSS and FGRSS, therefore being included in both tables.
