# Supplementary material for: Mapping gender role stress scales utilities: a scoping review approach
Source: Front Psychol. 2024 Sep 6;15:1436337. doi: 10.3389/fpsyg.2024.1436337 (PMC11414018; doi:10.3389/fpsyg.2024.1436337)
Supplement: Supplementary file 1 [file Data_Sheet_1.PDF]

Supplementary Material

Supplementary Figures and Tables

Table A1

Summary of the Results From the use of the FGRSS Through Time in Science

TOPIC	AUTHORS	MAIN FINDINGS
**Body Image**	Martz et al. (1995)	- Women high in FGRS evidenced a cardiovascular stress in response to a body image threat exam.<br>- Women who rigidly accept and attempt to fulfill the gender role challenge are vulnerable to experience distress.<br>- Women high in FGRS may experience more chronic stress.
	**Bekker & Boselie (2002)**	- Women with eating disorders experience more FGRS and higher stress level in general (vs. women without eating disorders). Especially due to the "fear of physical unattractiveness" dimension.<br>- Higher levels of recently experienced stress reported by the bulimic women were robust against controlling for feminine gender role stress (vs. control women).
	Kazmierczak & Goodwin (2011)	- Masculinity and androgyny were positively correlated with body image during pregnancy.<br>- Body image is a mediator between gender factors and self-esteem.<br>- Women that follow cultural norms set for female roles are prone to lower body image satisfaction.
	Khattab et al. (2022)	- FGRS is positively correlate with BDD in women with willingness to go under cosmetic surgery.<br>- FGRS is negatively correlate with life satisfaction in women.<br>- Higher BDD, FGRS and religiousness are predictors of the willingness to undergo cosmetic surgery (in Muslim women)
**Femininity / Femininity Ideology**	Davis et al. (2018)	- In African American women FGRS did not moderate the relationship between neither SBWI or TFI with perceived stress. But racial stress did.<br>- In African-American women, racial stress exacerbates the existing stress level especially when these women endorse SBWI.
	**Harrington et al. (2022)**	- FGRS and low femininity predict lower self-esteem in women.<br>- Women with higher FGRS consistently experience lower self-esteem (vs. women with lower FGRS).<br>- There´s a significant interaction between FGRS and weekly felt-femininity.
**Mental Health**	Tang & Chung (1997)	- Chinese women's psychosexual adjustment following either elective or postpartum sterilization was correlated with their fear of evaluation by others, family cohesion and adaptability, and preoperative psychological functioning.<br>- Chinese women that were quite satisfied with the postpartum sterilization; showed less physical, psychological, or sexual ill-health; and expressed infrequent reporting of regret and thoughts of reversal.
	**Efthim et al (2001)**	- Perceived gender role stress, in women, is a contributor to shame.<br>- FGRS is associated with shame-proneness and externalization; but it is NOT associated with guilt-proneness.<br>- The "being nurturance" and "emotional detachment" dimension of FGRS had a weaker association. Then, those may not be components of gender role stress that bring forth strong feelings of guilt and shame.<br>- These findings stand in contrast to body image literature.
	**López et al (2011)**	- In women, as gender role stress reduces its presence, the perfectionist personality shifts from adaptive to maladaptive.
	**Kazmierczak et al (2013)**	- Women have higher scores in borderline, histrionic and dependent personality (compared to men).<br>- Empathic concern is positively associated with stress in situations stereotypically identified as feminine.
	**Kargin et al (2021)**	- Female health students suffer less gender-role stress (vs. male students).<br>- In female health students, gender role stress decrease with an increase in age.<br>- There were no significance differences between socioeconomic level and gender-role attitudes in women.<br>- Relationship between gender roles, gender perception, and violence tendency of health school students found that female students had positive gender perceptions.<br>- Self-esteem decreased as gender-related stress increased.
	**Berry y Holloway (2022)**	- Black female college students are striving to meet the expectations and standards set forth by their gender-roles and are experiencing feminine gender-role stress.<br>- Black female college students suffer in silence in attempts to meet the expectations of others while maintaining the façade of strength, determination, and

		- The Strong Black Woman Ideology is specific to Black women's adherence to both traditional and nontraditional feminine norms, which is expected to be associated with stressful experiences.
	Kelmendi & Jemini-Gashi (2022)	- Women who experience lack of support are less satisfied and tend to be depressed.<br>- FGRS is positively associated with psychological distress.
**Work Context**	Tang & Lau (1996)	- Compared to men, women felt more stress in situation demanding feminine attributes.<br>- Individuals working in gender-typed professions experiment more gender role stress.<br>- Gender role stress and burnout associations were found only in gender-typed professions.<br>- FGRS was related to burnout dimensions for male professionals.
	**Bekker et al. (2001)**	- Women compared with men reported to have fewer executive tasks and possibilities in their jobs, and less participation and autonomy.<br>- Training effects were not affected by being male or female.<br>- Women suffered from more health and stress complaints.
	**Birze et al. (2020)**	- Findings indicate chronic forms of gendered workplace stress such as emotional labor, gender role stress and, posttraumatic stress each have differential associations with physiological responses to stressful events in the workplace
**Victimization**	Truman-Schram et al (2000)	- There's no statistical difference in scores of FGRS between women who leave and women who stay in an abusive relationship.<br>- FGRS subscales have a significant effect on the reports of global commitment.<br>- FGRS ("Fear of unemotional relationship") predicts relationship commitment.<br>- High scores in FGRS ("Fear of behaving assertively") predict women reporting what they like their partners more.
	Diaz-Aguado et al. (2022)	- Adolescents victims of IPV had lower self-esteem and more FGRS when they did not conform to sexist stereotypes, possibly leading them to exaggerate traditional female submission to male domination and violence.<br>- FGRS in victims correlate positively with: Subjective health complains (somatic and psychological), JMDV, Problematic use of internet and risky sexual online behavior among others.
**Parenting**	**Morse et al (2000)**	- First-time pregnant women experience distress/dysphoria during mid- and late pregnancy.<br>- The pattern of distress in women us characterized by a gradual increase in vulnerability from mid-pregnancy on.
	**Durkin et al. (2001)**	- Psychosocial functioning of couples during pregnancy is represented by anxiety, anger, gender role stress and affect.<br>- Gender role stress has a relation with mood during mid-pregnancy.<br>- For the women, practical support was important from family and emotional support from friends.<br>- In women concerned with the couple's own relationship functioning, the strongest variables loading on were (1) the sense of being cared by her partner, (2) the satisfaction with the support, and (3) her own assessment of the couple's relationship quality.
**Neurobiological differences**	**Lungu et al. (2015)**	- Greater prevalence of depression and anxiety in women (vs. men).<br>- Higher left amygdala activations in women.<br>- Women have a more purely affective (in-the-moment) brain response during negative emotion processing.<br>- Granger connectivity is negatively correlated with femininity → that sex-differences in emotion-related connectivity are not fully explain by hormonal factors, but also by social learning of stereotyped gender roles.
**Gender Equality**	Kurtulus & Bulut (2021)	- Gender Equality in women is NOT influence by: age levels, class level, education level of the father.<br>- Gender Equality in women is influence by: mother's educational level, GRS and FNE.<br>- More egalitarian thinking in women, the higher stress women get.

Note. FGRS =Feminine Gender Role Stress; BDD =Body dysmorphic disorders; SBWI =Strong Black Women Ideology; TFI =Traditional Femininity Ideology; IPV =Intimate Partner Violence; JMDV =Justification of Male Dominance and Violence; FNE =Fear of Negative Evaluation. Authors in red bold letters means that the article used both MGRSS and FGRSS.

Table A2

Summary of the Results From the use of the MGRSS Through Time in Science

TOPIC	AUTHORS	MAIN FINDINGS
**Body Image**	**Bekker & Boselie (2002)**	- Stress associated with masculinity emerged as a relevant stress for women with an eating disorder
	Abbas & Karadavut (2017)	- Likehood of having cosmetic surgery in men.<br>- Men’s boy image dissatisfaction is growing up recently → avoid appearing “girlie” to their peers.<br>- The currently idealized male form is complicated with the social and physiological dynamics of masculinity.
	Susansky & Dobrossy (2019)	- Levels of GRS are higher among overweight/obese men.<br>- Anxiety related to sexual performance, physical appearance, etc. causes a significantly high level of stress among males.
**IPV**	Eisler et al. (2000)	- Men high in MGRS reported more negative attributions and more negative affect (vs. low MGRS).<br>- More negative attributions and negative affect were reported for the woman's behavior in gender-relevant than gender irrelevant situations.<br>- Men high in MGRS made reliably more negative attributions about the woman's behavior (vs. low MGRS)<br>- Men high in MGRS reported reliably greater anger and irritation (vs. low MGRS).<br>- Men high in MGRS endorsed the use of verbal and physical aggression reliably more frequently (vs. low MGRS)
	Copenhaver et al. (2000)	- High-MGRS substance abusing men experience greater levels of anger (vs. low-MGRS substance-abusing men).<br>- High-MGRS substance abusing men were more likely to abuse substances as a means of coping with negative emotions such as anger.<br>- High-MGRS substance-abusing men did report significantly greater frequency of engaging in verbally abusive behaviors directed at their female partners.
	Franchina et al. (2001)	- High MGRS men made more negative attributions (vs. low MGRS) in gender relevant situations.<br>- High and low MGRS men differed in their attributions about threat from a female partner.<br>- Significant interaction of MGRS by Female Threat.<br>- High MGRS men are more susceptible to appraising challenges to their masculinity ideology as stressful (vs. low MGRS).<br>- Female partner's behavior threatens masculinity ideology → high MGRS men appraise that threat as more stressful and, make more negative attributions about the woman (vs. low MGRS).<br>- The endorsement of verbal aggression by high MGRS may depend on the combined effects of female threat and masculine gender relevance as sources of stress.
	Jakupcak et al. (2002)	- MGRS scale and GRC scale do not represent a single construct.<br>- The cognitive factor of masculine ideology composite (by itself) did not explain a significant portion of the variance of aggression and violence.<br>- Subjective reports of “stress” may reflect men’s frequent experience of physiological states when their male gender norms are violated.<br>- Physically aggressive men reported higher levels of internal arousal while arguing with their female partners than did nonviolent men.<br>- At high levels of masculine ideology, gender role stress significantly predicted men’s aggression and violence.
	Jakupcak (2003)	- Cognitive and experiential factors of masculinity (ideology or masculine gender role stress) are not the primary factors that lead men to perpetrate aggression and violence.<br>- Men’s fear of anxiety and feelings of sadness were positively related to self-reports of aggression and violence.<br>- Men’s fear of positive feelings was significantly associated with men’s reported relationship violence.<br>- MGRS and men’s fear of emotions positively predict men’s reports of aggression and violence.
	Moore & Stuart (2004)	- Masculine gender-relevant situations produced more negative attributions, anger, negative affect, and verbal aggression than did masculine gender-irrelevant situations.<br>- High MGRS men reported greater anger, negative attributions, and verbal aggression.<br>- high MGRS men may be particularly sensitive to appraising any intimate conflict situations as threatening their understanding and ability to enact masculine gender role imperatives.
	Mahalik et al. (2005)	- Gender role stress partially mediated the relationship between one type of insecure attachment (i.e., fearful attachment) and controlling behaviors in a sample of men who batter.

Anderson & Anderson (2008)	- Hostility toward women specifically predicts heightened aggression against women.<br>- Individual differences predict aggression against women in general, not just sexual aggression.
Gallagher & Parrott (2011)	- Adherence to the masculine hegemonic norms (antifemininity, status and toughness) was associated with hostility toward women.<br>- Adherence to the status and antifemininity norms was not directly associated with men's hostility toward women.<br>- Adherence to the status and antifemininity norms was indirectly related to hostility toward women through MGRS.<br>- Adherence to the toughness norm has a positive directly association with hostility towards women, without any moderating effect from MGRS.
McDermott & Lopez (2013)	- Gender role stress partially mediate the relationships between attachment dimensions and IPV acceptance attitudes.
Reidy et al (2014)	- Masculine gender role conformity was associated with IPV perpetration.<br>- MGRS predicted the historical perpetration of the three assessed forms of IPV.<br>- In men identifying as heterosexual, the more they endorsed sexual attraction or sexual behaviors with other men in their past, the more they endorsed acts of violence in their intimate relationships with women.
Smith et al (2015)	- Adherence to the antifemininity norm and the tendency to experience stress when in subordinate positions to women (i.e., high-MGRS) were indirectly related to sexual aggression perpetration via adherence to the sexual dominance norm (i.e., to be sexually aggressive and/or coercive toward an intimate partner in order to maintain their need for dominance within their intimate relationship).
Lisco et al (2015)	- Adherence to the toughness and antifemininity norms, but not the status norm, is indirectly and positively associated with the perpetration of physical aggression toward female intimate partners via MGRS.<br>- MGRS mediates these indirect effects only among men who engage in heavy episodic drinking.
Leone et al. (2016)	- Men's adherence to traditional masculinity norms is associated with their confidence to intervene in male-to-female sexually aggressive events.<br>- Adherence to the status and toughness norms is associated with bystander efficacy via bystander decision-making. Adherence to the antifeminine norm is directly associated with less confidence for intervention.<br>- High-MGRS men tend to appraise behaviors or situations that deviate from the traditional male gender role as adverse.<br>- The association between adherence to certain masculine norms (i.e., status) and bystander decision-making is dependent upon this gender-relevant context.
McDermott et al. (2017)	- MGRS was related to attitudes accepting of psychological, physical, and sexual dating violence in both the male and female samples, as well as positively related to acceptance of physical and sexual violence.<br>- Masculine gender role conflict may be more closely associated with attitudes promoting violence against women than other domains, especially SPC and CBWFR.
Zapata-Calvente et al. (2019)	- Implicit associations of own partner with violence as well as hostility were generally high but did not correlate strongly with IPV proclivity measures.<br>- IPV Proclivity measures were positively predicted by hostile sexism, MGRS, and short-term mating orientation.<br>- Log-term mating orientation negatively predicted IPV proclivity.
Harrington et al. (2021)	- Men who perceived they had lower relationship power reported greater physical aggression toward their partner during conflict and sexual rejection, but only when they experience stress within situations that can threaten men's traditional masculine identity (i.e., were higher in MGRS).<br>- Low relationship power can threaten men's masculine identity and aggression may be used by men to restore relationship power and demonstrate their masculine identity.<br>- Greater MGRS significantly predicted verbal aggression independent of relationship power, whereas greater MGRS only predicted physical aggression when men had lower relationship power.
Merino et al. (2021)	- MGRS, mediates the relationship between MADVAW and the justification of such violence.<br>- At the beginning of adolescence, it is MGRS related to situations of subordination to women that is a much better predictor of MADVAW than low self-esteem.
Diaz-Aguado & Martinez-Arias (2022)	- In the four variables related to the underlying mentality behind violence against women (JMDVAW, JVCR, MGRS-WS, and MGRS-II), the group of abuse in the three contexts (dating violence online, offline and sexual harassment) registered significantly higher scores than the group associated to sexual harassment online, which in turn scored higher than the non-violent group. Especially the first one.<br>- As expected, the group corresponding to abuse in the three contexts revealed low levels of self-esteem, and this variable has a significant predictive value for membership of this group.

	Chan & Poon (2023)	- Ostracism, in men, predict anxiety and depressive symptoms through masculine gender role stress and intimate partner violence perpetration.
**Sexual harassment/ coercion**	Moore et al (2008)	- Gender role stress regarding failure to perform in work and sexual domains was the only factor associated with psychological aggression.<br>- Gender role stress regarding appearing physically fit and not appearing feminine was the only factor associated with sexual coercion.<br>- Gender role stress regarding intellectual inferiority was the only factor associated with injury to partners.
	Mellon (2013)	- A positive correlation between levels of stress specifically related to subordination to women and levels of sexual harassment proclivity.<br>- Self-reported harassment proclivity scores were also positively correlated with scores on empirically derived dimensions of masculine gender role stress that were not thematically related (or were at most weakly related) to women's gender role violations.<br>- Thus, quid pro quo sexual harassment in men might be motivated by women's gender role violations.<br>- Threat stimuli produced by other forms of masculine gender role violations might occasion uninvited but "manly" sexual behavior as well—aggression that might also be directed at women who conform to traditional feminine gender role expectations.
**Well-being**	Eisler et al (1988)	- Men experience more MGRS than women.<br>- MGRS is a construct distinct from masculinity<br>- Scores on the MGRS questionnaire predicted negative psychosocial and somatic consequences.
	Lash et al (1990)	- MGRS is related to increased systolic blood pressure responses to the cold-pressor when it is presented in a way designed to elicit masculine performance challenge, but not when masculine challenge is minimized.
	Saurer & Eisler (1990)	- High-MGRS men were found to be less expressive, were rated by observers as significantly more anxious when expressing tender emotion, were found to be the less expressive and rated themselves as feeling more hostile (vs. Iow-MGRS).<br>- The two MGRS groups (high and low) did not differ significantly on their social network size
	Eisler & Blalock (1991)	- Excessive commitment to masculine gender role values may both produce gender role stress and lead to the development of inflexible and maladaptive coping patterns.
	Mahalik & Lagan (2001)	- Gender role conflict factors and gender role stress factors were related to religious orientation and spiritual well-being.<br>- Being rigid in terms of traditional masculinity and being stressed about living up to traditional roles related to less intrinsic religiosity and spiritual well-being.<br>- Gender role conflict and stress may play different roles in each groups' religiosity and spiritual wellbeing (seminarians vs. non-seminarians men).
	Consenzo et al (2004)	- Some men are susceptible to appraise masculine relevant information as stressful and that this appraisal has physiological and behavioral consequences.<br>- High MGRS men exhibited significantly greater increases in systolic blood pressure under masculine gender-relevant instructions (vs. under masculine gender-irrelevant instructions).<br>- Under masculine GR instructions, the performance of high MGRS men on the serial subtraction task was impaired relative to that of low MGRS men.<br>- MGRS Scale is useful for identifying men with a propensity to become stressed when they appraise certain situations as challenges to their masculinity.
	Morrison (2012)	- A significant interaction between conformity to masculine norms and symptoms of possible posttraumatic stress disorder in the prediction of health behaviors.<br>- The Emotional Inexpressiveness subscale of the MGRS was uniquely associated with probable PTSD symptom severity.
	Arrindell et al (2013)	- Societies that put strong emphasis on men being committed to culturally accepted models of masculinity had higher mean national levels of MGRS than those that put less emphasis on such commitment.<br>- High masculinity societies reported higher levels of MGRS than "soft" (low masculinity) societies.<br>- Higher levels of country-level instrumentality predicted, independent of masculinity scores, lower country levels of MGRS.
	Sloan et al (2015)	- MGRS is related with poor mental and physical health and detrimental health habits.<br>- High levels of MGRS and male role norm adherence predicted worse health behaviors for both men and women (i.e., lower levels of positive health behaviors and higher levels of negative health behaviors), although these relationships were more numerous and stronger for men.
	Kantar & Yalcin (2023)	- self-stigma and self-compassion serially mediate the relationship between masculine gender role stress and attitudes toward seeking psychological help.
**LGTBIQ+ Community &**	Crawford et al (2002)	- AAGBM identified in the Integration Mode of Racial-Ethnic Sexual Identity Acculturation (i.e., individuals possessing very positive self-identification as being African American and gay) reported experiencing higher levels of self-esteem, HIV prevention self-efficacy,

**Anti-LGTBIQ+ Community**		stronger social support networks, greater levels of life satisfaction, and lower levels of male gender-role and psychosocial distress (vs. other modes).<br>- AAGBM identified in the Marginalization Mode of Racial-Ethnic Sexual Identity Acculturation (i.e., individuals possessing negative self-identification as being African American and gay) reported experiencing the greatest amount of psychosocial distress and the lowest levels of self-esteem, social support, and life satisfaction.<br>- Higher levels of psychosocial distress and gay identity, as well as lower levels of life satisfaction, are associated with higher levels of sexual risk-taking among this sample of AAGBM.
	Zamboni & Crawford (2007)	- Racial discrimination, gay bashing, and male gender role stress may increase the chances that sexual problems develop among African-American gay/bisexual men.<br>- Perceived experiences with discrimination based on race/ethnicity and sexual orientation create stress and, adversely affect sexual functioning.<br>- Male gender role stress adversely affects sexual functioning.
	Parrot et al (2008)	- Sexual prejudice fully mediated the association between status and anger in response to gay men.<br>- MGRS partially mediated the effect of antifemininity on anger in response to gay men.<br>- Male gender role norms, particularly the antifemininity norm, are strongly associated with anger in response to gay men and that sexual prejudice and MGRS are important mediators of these associations.
	Parrott (2009)	- Adherence to the antifemininity norm elicits anger and aggression toward gay men among men high in masculine gender role stress, that adherence to the antifemininity norm is fundamental to the enforcement of gender norms via aggression toward gay men.
	Parrott et al (2011)	- Strong adherence to traditional norms of masculinity, particularly the status and antifemininity norms, is a key determinant of aggression toward sexual minorities and sexual prejudice accounts for these effects.<br>- Endorsement of the antifemininity norm exerted the strongest direct and indirect effects on aggression toward male and female sexual minorities. In particular, the direct effect of antifemininity on aggression was about three times larger than the indirect effect mediated by sexual prejudice and masculine gender role stress.
	Vincent et al (2011)	- MGRS, sexual prejudice, and antigay anger collectively facilitate antigay aggression.<br>- MGRS is positively associated with frequency of perpetrating acts of antigay aggression.<br>- Men high in MGRS respond aggressively to masculinity threats, regardless of whether the threat originates from women or gay men.
	Daboin et al. (2015)	- Acceptance of the traditional male role norms of status and toughness, religious fundamentalism, and sexual prejudice against lesbians and gay men is higher among Black heterosexual men (vs. white heterosexual men).<br>- The traditional male role norm of status is more positively associated with sexual prejudice toward gays and lesbians among Black heterosexual men (vs. white heterosexual men).
**Masculinity**	Jakupcak et al (2003)	- Traditionally masculine men report lower overall levels of affect intensity.<br>- MGRS was not significantly related to affect intensity.<br>- Masculine ideology was positively associated with men's global fear of emotions, fear of anger, positive emotions and depressed mood.
	Jakupcak et al (2005)	- Masculinity is positively related with fear of emotions, and proneness to shame and external expression of anger and hostility and aggression.<br>- Masculine gender role socialization contributes to men's aggressive tendencies by limiting alternative emotional expressions and/or interfering with men's ability to tolerate vulnerable feelings.
	Cohn & Zeichner (2006)	- High-masculinity identity men react more impulsive and aggressively toward another (vs. low-masculinity identity men)<br>- In high-MGRS men, masculine identity would exert a significant influence on the prediction of aggressive behavior (vs. low-MGRS men).
	Yeung et al (2015)	- ECMN was associated with poorer psychological well-being among Chinese men.<br>- Men low-AEE have a stronger negative association between ECMN and psychological well-being (vs. men high-AEE).
	Chadwick & Van Anders (2017)	- Women's orgasms function as a masculinity achievement for men.<br>- Men felt most masculine after imagining that a female partner orgasmed with them (vs. she did not). This effect was exacerbated for men with high MGRS.<br>- High-MGRS men felt more masculine when a woman orgasmed and less masculine when she did not (vs. low-MGRS men).
	Gebhard et al (2019)	- There's link between threatened masculinity shame-related responses and a physical aggression.<br>- Men who are prone to general shame are also more prone to experience shame related to threatened masculinity.
	Booth et al (2019)	- Men who are likely to appraise violating their gender roles as stressful (high MGRS) are also likely to believe that seeking counseling would have a negative impact on their self-worth (self-stigma).<br>- Self-compassion was negatively associated with MGRS.

		- Self-coldness was positively associated with MGRS.<br>- Self-compassion was a significant negative predictor of self-stigma.<br>- High self-compassion moderated the associations between MGRS and self-stigma.<br>- Low self-coldness moderated the association between MGRS and self-stigma.
	Scaptura & Boyle (2022)	- Acceptance threat, threat to an individual man's sense of masculinity, and status threat and the belief that societal changes disadvantage men as a group are positively associated with both men's attraction to guns and their aggressive reactions to perceived disrespect.<br>- The effect of acceptance threat is amplified when a strong sense of status threat is also present, including attraction to guns and aggressive reaction to disrespect.<br>- The racial and classed intersections amplify beliefs of status and acceptance threat for white men, channeling these threats into aggression and attraction to guns.
**Parenting**	**Morse et al (2000)**	- The couple's pregnancy invokes in the men anticipated fears of work disruption when the baby arrives and/or loss of the sexual relationship due to reluctance and discomfort by the female partner.<br>- Men first pregnancy → the work- and sex performance related fears may have masked perceived threats to the male identity in some men, and/or uncertainties about their approaching new role as a father.
	**Durkin et al (2001)**	- Psychosocial functioning of couples during pregnancy is represented by anxiety, anger, gender role stress, and affection.<br>- In the men, relationship functioning was characterized by the sense of being cared for by his partner.<br>- For the men, the family seemed have greater importance in the social support dimension than friends.
	DeFranc & Mahalik (2002)	- Men's own gender role conflict and stress were related to lesser levels of parental attachment and greater degree of psychological separation from both father and mother.<br>- Androgynous men to have more positive relationships with parents, particularly with fathers, than stereotypically masculine men.<br>- Men's estimates of their fathers' gender role strain would be related to less attachment and greater psychological separation from both parents.
	Buist et al. (2003)	- Most men deal well with the transition to fatherhood and their anxieties resolve over time, infrequently reaching a level of clinical illness.<br>- Men anxieties may be missed antenatally because the greatest amount of attention is given to the pregnant woman.<br>- For a small number of men, the association of distress with lower marital satisfaction and lower paternal attachment has implications for the whole family.<br>- In men, gender role stress is closely associated with postnatal depression.<br>- Distressed men, were more likely to be anxious about aspect of their social role.
	Casselman & Rosenbaum (2014)	- Fathers' beliefs and actions influence their sons' aggression, and thus provides an important contribution to the intergenerational violence.<br>- Abusive men might realize their female-directed violence could be related to endorsing their fathers' hypermasculine beliefs and observing their fathers' spousal abuse as boys.<br>- Sons endorse more easily hypermasculine beliefs similar to fathers when fathers are loving and accepting them.<br>- Compared to highly masculine males with rejecting fathers and low self-esteem, some highly masculine males with accepting fathers might experience less masculine gender role stress due to their strong self-confidence but are still aggressive because that is part of their belief system regarding how a man should behave.
	Chabra et al. (2022)	- MGRS as a risk factor, with the quantitative analysis confirming MGRS being the greatest predictor for paternal perinatal depression and anxiety.<br>- Men who strongly adhered to traditional masculine norms may find it difficult to transition to an egalitarian fatherhood and, hence, experience high levels of MGRS.<br>- Marital distress was identified as a risk factor for paternal perinatal depression and anxiety.<br>- According to traditional masculine gender norms, fathers who identify with traditional gender roles may place more expectations in their work responsibilities than their home responsibilities and often experience work–family conflict.
**Substance Abuse**	Isenhart (1993)	- Sample of inpatient alcohol abusers →MGRSS assessed stress levels associated with inadequate performance, sexual inadequacy, engaging in traditionally feminine behavior, and job inadequacy.<br>- Higher MGRSS scores (especially in Sexual Inadequacy and Inadequate Performance subscales) obtained higher scores on alcohol abuse.
	McCreary et al (1999)	- For men, traditional attitudes led to more alcohol consumption, agentic traits protected them from experiencing alcohol-related problems, and experiencing MGRS was a risk factor for these problems.
	McDermott et al (2010)	- There´s an association between MGRS and PTSD symptom severity among male crack/cocaine dependent patients in residential substance abuse treatment.<br>- MGRS was found to be significantly associated with posttraumatic stress disorder symptom severity when controlling for other variables previously found to be associated with posttraumatic stress disorder.

	Klingemann & Gómez (2010)	- Patients (men in inpatient alcohol programs) show significantly lower values on the masculinity subscale and lower values on the femininity subscale (vs. control group).<br>- Patients who acknowledge men-specific treatment needs suffer significantly more from gender role stress and problems with sexuality and fatherhood than patients who are not aware of masculinity issues.<br>- Men who did not think that masculinity issues were relevant for therapy showed more traditional gender role orientations (vs. men in who did see the relevance of men-specific topics in addiction therapy).
	Gallagher y Parrot (2016)	- Men with lower, but not higher, masculine gender role stress who received the intervention manipulation, relative to control, enacted significantly less alcohol-related physical aggression toward women .
	Leone et al (2022)	- Men higher in MGRS who were intoxicated (alcohol) were more likely than sober men to select the sexually explicit film when the woman was intoxicated and less likely than sober men to select the sexually explicit film when the woman was sober.
**Mental health**	Arrindell et al (1993)	- Biological sex emerged as a consistent predictor of fears.<br>- Masculinity and masculine gender role stress level are also predictive of higher fear levels.
	McCreary et al. (1996)	- MGRS was related to depression, hostility, and anxiety, but to the same degree for both men and women.<br>- If MGRS is a valid construct, then researchers need to explore the ways in which this type of stress can negatively affect men, but not women → looking for more relevant male-oriented outcomes.
	Ragsdale et al. (1996)	- (In war-related posttraumatic stress disorders inpatients treatment effectiveness) → treatment utilizing adventure-based counseling, psychoeducational, and psychodrama therapeutic modalities was effective e in reducing feelings of hopelessness, guilt, shame, loneliness and emotional isolation.<br>- Indices of psychological functioning (including interpersonal skills, MGRS, anxiety, anger, and PTSD symptomatology) did not change significantly in response to treatment.
	**Efthim et al. (2001)**	- Perceived gender role stress, in men, is a contributor to shame.<br>- A tendency for men to be stressed when expressing vulnerable feelings and when women are in charge is associated with the defensive strategy of externalization and a tendency not to experience guilt.<br>- Men who are strongly committed to gender schemas may tend to manage emotional distress by directing it outward and disowning it rather than internalizing it in the form of conscious guilt.<br>- MGRS in men was linked to guilt, although this was also mixed with a tendency to blame others and to experience shame.<br>- MGRS was associated with shame-proneness.
	Arrindell et al (2003)	- MGRS subscales (Physical inadequacy, Emotional inexpressiveness, Subordination to women, Intellectual inferiority, and Performance failure) predicted Agoraphobic fears, Blood-Injury fears, Social fears, and Obsessional checking and washing compulsions distinctively.<br>- Intellectual inferiority subscale was the strongest predictor of Social fears.
	Jakupcak et al. (2006)	- (in male veterans) → MGRS was significantly and positively associated with alexithymia and significantly and negatively related to social support.<br>- MGRS was not associated with PTSD symptom severity or fear of emotional states.
	**López et al (2011)**	- In men, as gender role stress increases its presence, the perfectionist personality shifts from adaptive to maladaptive.
	**Kazmierczak et al. (2013)**	- Men have higher scores in Schizoid and antisocial personality (compared to women).<br>- MGRS plays a “protective role” against emotional dysfunction associated with personal distress.
	**Kargin et al. (2021)**	- (In health students) →Gender-related stress in men is higher than in women.<br>- Relationship between gender roles, gender perception, and violence tendency of health school students and found that male students had negative gender perceptions.<br>- It was found that there was a statistically significant relationship between the self-esteem total score of male students and their mother's educational status.<br>- Self-esteem decreased as gender-related stress increased.
	**Berry y Holloway (2022)**	- Black male college students report conforming to gender-role norms and experiences of stress, to a lesser degree compared to Black female college students.<br>- Black men often experience competing masculinities-one culture of masculinity from a Eurocentric perspective.
	**Harrington et al (2022)**	- In women higher levels of MGRS were associated with lower self-esteem. This relationship was not found in men.<br>- There was no significant interaction between MGRS and weekly felt-femininity.
**Neurobiological differences**	**Lungu et al (2015)**	- Granger connectivity from right amygdala to dmPFC (stronger in men) was negatively correlated with subjective ratings of high negative images and positively correlated with testosterone levels → that sex-differences in emotion-related connectivity are not fully explain by hormonal factors, but also by social learning of stereotyped gender roles.

		- Men have a more evaluative, in-the-moment, brain response during negative emotion processing.
**Work Context**	Watkins et al (1991)	- Men obtained significantly higher scores than women on a MGRS scales.<br>- Women with elevated MGRS Scale scores, experienced undesirable outcomes as much as their male counterparts → Type A behavior, hostility, personal loss, life dissatisfaction, and elevated systolic and diastolic blood pressure.<br>- MGRS Scale scores were related to unfavorable perceptions of the workplace among women but to favorable perceptions among men.<br>- Women with elevated MGRS Scale scores may have been experiencing this dilemma: individuals' adoption of both masculine and feminine gender roles may be problematic in that they now have not one, but two, sources of potential inadequacy.
	**Tang & Lau (1996)**	- MGRS was related to emotional exhaustion and depersonalization for both male and female professionals.<br>- Gender role stress and burnout associations were found only in gender-typed professions.
	**Bekker et al. (2001)**	- Working in gender-typed professions experiment more gender role stress.<br>- Men, compared with women, reported to have higher executive tasks and possibilities in their jobs, and more participation and autonomy.
	**Birze et al. (2020)**	- Findings indicate chronic forms of gendered workplace stress such as emotional labor, gender role stress and, posttraumatic stress each have differential associations with, and predict physiological responses to stressful events in the workplace

Note. GRS= Masculine Gender Role Stress; GAM= General Aggression Model; JMDVAW= Justification of male dominance and violence against women; JVCR= Justification of violence as conflict resolution; MGRS-WS= Masculine gender role stress-women subordination; MGRS-II= Masculine gender role stress-intellectual inferiority; GRC= Gender Role Conflict; GRCS= Gender Role Conflict Scale; SPC= Success, power and Competition from GRCS; CBWFR= Conflict Between Work and Family Relations from GRCS; MADVAW= Male Adolescent Dating Violence Against Women; AAGBM= Afro-American Gay and Bisexual men; ECMN= Emotional Control Masculine Norm; AEE= Ambivalence over Emotional Expression. Authors in red bold letters means that the article used both MGRSS and FGRSS

Table A3

Summary of the Results From the Authorship Analysis

	First author is a man	First author is a woman	More men in the authorship	More women in the authorship	Only men in the authorship	Only women in the authorship
**MGRS is the only one used**	41	21	34	12	23	5
**FGRS is the only one used**	1	8	1	4	0	2
**BOTH scales are used**	5	9	2	10	0	7
**SUBTOTAL**	47	38	36	26	23	14
**TOTAL**	85 + 2 with unknown or non-binary first authorship		62 + 25 articles with an equal number of male and female authors		37 + 50 articles with mixed authorship of men and women	
